# Supplementary material for: Substituent-Dependent Divergent Synthesis of 2-(3-Amino-2,4-dicyanophenyl)pyrroles, Pyrrolyldienols and 3-Amino-1-acylethylidene-2-cyanopyrrolizines via Reaction of Acylethynylpyrroles with Malononitrile
Source: Molecules. 2022 Dec 3;27(23):8528. doi: 10.3390/molecules27238528 (PMC9737003; doi:10.3390/molecules27238528)
Supplement: Supplementary file 1 [file molecules-27-08528-s001.zip › molecules-2073606-supplementary.pdf]

Supporting Information

Substituent-dependent divergent synthesis of 2-(3-amino-2,4-dicyanophenyl)pyrroles, pyrrolyldienols and 3-amino-1-acylethylidene-2-cyanopyrrolizines via reaction of acylethynylpyrroles with malonitrile

Maxim D. Gotsko, Ivan V. Saliy, Igor' A. Ushakov,
Lyubov N. Sobenina, Boris A. Trofimov*

A.E. Favorsky Irkutsk Institute of Chemistry, Siberian Branch of the Russian Academy of Sciences, 1 Favorsky St., Irkutsk 664033, Russian Federation

**Corresponding author: Tel.: 7 (3952) 51-14-31; fax 7 (3952) 41-93-46; e-mail: boris_trofimov@irioch.irk.ru*

Table of contents

General information 3

Synthesis of 2-(3-amino-2,4-dicyanophenyl)pyrroles (3a-m), pyrrolyldienols (4a-c) and pyrrolizines (6b,c) (General Procedure) 3

Synthesis of 3-amino-1-acylethylidene-2-cyanopyrrolizine (6a) 9

The NMR spectra: 10

The 2D NMR spectra: 50-58

General information

IR spectra were obtained with a Bruker Vertex 70 spectrometer (400–4000 cm–1, KBr). 1H (400.13 MHz), 13C (100.6 MHz) spectra were recorded on a Bruker DPX-400 spectrometer at ambient temperature in CDCl3 solutions and referenced to CDCl3 (residual protons of CDCl3 in 1H NMR δ = 7.27 ppm; 13C NMR δ = 77.1 ppm) and DMSO-d6 (residual protons of DMSO-d6 in 1H NMR δ = 2.50 ppm; 13C NMR δ = 39.52 ppm). The assignment of signals in the 1H NMR spectra was made using COSY and NOESY experiments. Resonance signals of carbon atoms were assigned based on 1H- 13C HSQC and 1H-13C HMBC experiments. The C, H, N, S microanalyses were performed on a Flash EA 1112 CHNS-O/MAS analyzer. Clorine was determined by mercurimetric titration. Melting point (uncorrected) was determined on a Kofler micro hot-stage apparatus.

Malononitrile **2**, KOH and MeCN are commercial products. Acylethynylpyrroles was prepared according to the procedure reported[1].

Synthesis of 2-(3-amino-2,4-dicyanophenyl)pyrroles (3a-m), pyrrolyldienols (4a-c) and pyrrolizines (6b,c) (General Procedure)

The suspension of malononitrile **2** (132 mg, 2 mmol) and KOH·0.5H_2O (130 mg, 2 mmol) in acetonitrile (15 mL) was stirred at 20-25 °C for 30 min. Then the reaction mixture was cooled to 0 °C, the 2-acylethynylpyrrole 1 (1 mmol) in acetonitrile (5 mL) was added dropwise to a reaction mixture within 10 min. Reaction mixture was stirred at 0 °C for 2 h and then was diluted with water (40 mL), extracted with diethyl ether (4 x 10 mL). Extracts were washed with water and dried over Na_2SO4. The residue, after removing solvent, was fractionated by column chromatography (A*l2*O*3*, *n*-hexane : diethyl ether, 1 : 1) to afford the pyrroles **3a-m**. Under analogous conditions, pyrrolyldienols **4a-c** are formed from 2-acyletinylpyrroles **1n-p** and malononitrile **2**. They are isolated by filtering the formed precipitates by diluting the reaction mixtures with water (1:3). Pyrrolyldienols **4b,c** are formed with additive of the keto form. In the course of isolation and drying of products 4b,c, the corresponding pyrrolizines **6b,c** are formed from them.

*3-Amino-5-(1-methyl-1H-pyrrol-2-yl)-[1,1'-biphenyl]-2,4-dicarbonitrile (**3a**).*

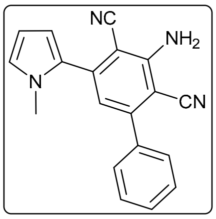

Yield 233 mg (78%); yellow solid, m.p. 213-215 °C. R_f = 0.68. [Found: C, 76.6; H, 4.86; N, 18.96%. $C_{19}H_{14}N_4$, requires C, 76.49; H, 4.73; N, 18.78%. ν_{max} (KBr) 3471, 3356, 3240, 2927, 2853, 2214, 2161, 1640, 1585, 1572, 1550, 1498, 1463, 1419, 1380, 1289, 1143, 1076, 1032, 959, 909, 733, 700, 633, 496. ^{1}H NMR (400.13 MHz, DMSO-d_6): δ 7.62-7.61 (m, 2H, *o*-Ph), 7.54-7.51 (m, 3H, *m,p*-Ph), 7.01-6.98 (m, 1H, H-5, pyrrole), 6.75 (s, 1H, CH, aniline), 6.74 (br. s, 2H, NH_2), 6.46-6.45 (m, 1H, H-3, pyrrole), 6.17-6.15 (m, 1H, H-3, pyrrole), 3.65 (s, 3H, CH_3). ^{13}C NMR (100.6 MHz, DMSO-d_6): δ 154.1, 149.3, 140.9, 137.4, 129.3, 128.9, 128.6 (2C), 128.5 (2C), 126.6, 118.5, 116.2, 116.1, 112.2, 107.9, 94.4, 93.2, 34.9.

*3-Amino-5-(4-ethyl-5-propyl-1H-pyrrol-2-yl)-[1,1'-biphenyl]-2,4-dicarbonitrile (**3b**)*

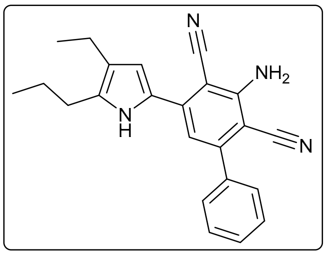

Yield 266 mg (75%); yellow crystals, m.p. 118-120°C; R_f = 0.66. [Found: C, 78.14; H, 6.40; N, 15.91%, $C_{23}H_{22}N_4$, requires $C_{23}H_{22}N_4$: C, 77.94; H, 6.26; N, 15.81%. ν_{max} (KBr) 3473, 3357, 3239, 2962, 2928, 2871, 2208, 2156, 1959, 1633, 1587, 1571, 1541, 1490, 1459, 1329, 1274, 1208, 1176, 1139, 1075, 1041, 1018, 908, 858, 820, 772, 731, 700, 649, 503 cm^{-1}. ^{1}H NMR (400.13 MHz, $CDCl_3$): δ 8.91 (s, 1H, NH), 7.56-7.53 (m, 2H, *o*-Ph), 7.49-7.47 (m, 3H, *m,p*-Ph), 6.92 (s, 1H, CN-Ph), 6.85 (d, *J* = 2.8 Hz, 1H, H3 of pyrrole), 5.27 (s, 2H, NH_2), 2.63-2.57 (m, 2H, CH_2), 2.45 (q, *J* = 7.6 Hz, 2H, CH_2), 1.71-1.62 (m, 2H, CH_2, CH_2), 1.19 (t, *J* = 7.5 Hz, 3H, CH_3), 0.99 (t, *J* = 7.3 Hz, 3H, CH_3). ^{13}C NMR (100.6 MHz, $CDCl_3$): δ 154.0, 149.7, 139.3, 138.0, 134.6, 129.6, 128.9 (2C), 128.4 (2C), 125.2, 125.0, 118.2, 116.7, 115.5, 113.7, 91.3, 88.0, 28.2, 23.0, 18.9, 15.6, 14.0.

*3-Amino-5-(4,5,6,7-tetrahydro-1H-indol-2-yl)-[1,1'-biphenyl]-2,4-dicarbonitrile (**3c**)*

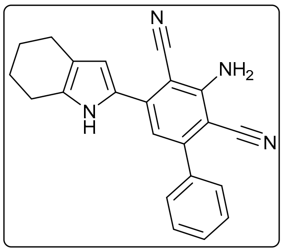

Yield 244 mg (72%); yellow crystals, m.p. 175-177°C; R_f = 0.67. [Found: C, 77.86; H, 5.55; N, 16.38%, $C_{22}H_{18}N_4$, requires $C_{24}H_{15}ClN_4$: C, 78.08; H, 5.36; N, 16.56%. ν_{max} (KBr) 3468, 3348, 3241, 2924, 2852, 2361, 2209, 1637, 1570, 1544, 1489, 1460, 1362, 1270, 1205, 1144, 1126, 1012, 931, 897, 851, 805, 771, 699, 637, 505 cm^{-1}. ^{1}H NMR (400.13 MHz, DMSO-d_6): δ 11.21 (s, 1H, NH), 7.63-7.60 (m, 2H, *o*-Ph), 7.54-7.51 (m, 3H, *m,p*-Ph), 7.01 (s, 1H, Ph-CN), 6.91-6.90 (m, 1H, pyrrole), 6.47 (s, 2H, NH_2), 2.61-2.57 (m, 2H, CH_2-7), 2.50-2.46 (m, 2H, CH_2-4), 1.78-1.74 (m, 2H CH_2-5,), 1.71-1.64 (m, 2H, CH_2-6). ^{13}C NMR (100.6 MHz, DMSO-d_6): δ 154.8, 149.1, 139.5, 138.0, 133.0, 129.2, 128.5 (2C), 128.4 (2C), 124.9, 119.0, 117.4, 116.6, 114.0, 111.2, 90.0, 87.9, 23.2, 22.7, 22.4, 22.4.

*3-Amino-5-(5-phenyl-1H-pyrrol-2-yl)-[1,1'-biphenyl]-2,4-dicarbonitrile (**3d**)*

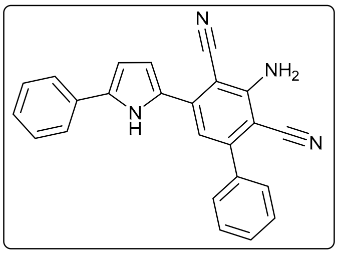

Yield 303 mg (84%); yellow crystals, m.p. 82-84°C; R_f = 0.66. [Found: C, 80.08; H, 4.52; N, 15.63%, $C_{24}H_{16}N_4$, requires $C_{23}H_{22}N_4$: C, 79.98; H, 4.47; N, 15.55%. ν_{max}(KBr) 3456, 3357, 3242, 3061, 2923, 2852, 2361, 2253, 2211, 1634, 1577, 1546, 1476, 1460, 1424, 1385, 1298, 1262, 1216, 1077, 1058, 856, 758, 700, 497 cm^{-1}. ^{1}H NMR (400.13 MHz, $CDCl_3$): δ 9.49 (s, 1H, NH), 7.59-7.56 (m, 4H, *o,m*-Ph), 7.50-7.49 (m, 3H, *o,p*-Ph), 7.44-7.40 (m, 2H, *m*-Ph), 7.32-7.28 (m, 1H, H*p*-Ph), 7.04-7.03 (m, 1H, H-3 of pyrrole), 7.02 (s, 1H, CN-Ph), 6.66-6.64 (m, 1H, H-4 of pyrrole), 5.31 (s, 2H, NH_2). ^{13}C NMR (100.6 MHz, $CDCl_3$): δ 153.9, 150.0, 138.9, 137.8, 136.9, 131.3, 129.8, 129.3 (2C), 129.0 (2C), 128.4 (2C), 128.3, 127.8, 124.5 (2C), 118.2, 116.4, 116.1, 115.1, 108.8, 92.4, 88.7.

*3-Amino-5-(5-(4-chlorophenyl)-1H-pyrrol-2-yl)-[1,1'-biphenyl]-2,4-dicarbonitrile **(3e)***

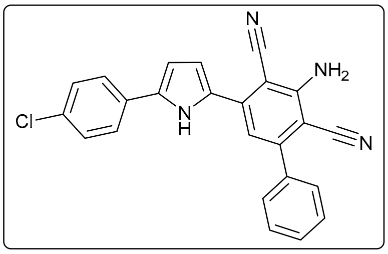

Yield 316 mg (80%); yellow crystals, m.p. 236-238°C; R_f = 0.68. [Found: C, 73.20; H, 3.84; N, 14.19%, $C_{24}H_{15}ClN_4$, requires $C_{24}H_{15}ClN_4$: C, 73.00; H, 3.83; Cl, 8.98; N, 14.19%. ν_{max}(KBr) 3464, 3446, 3353, 3240, 2214, 1645, 1584, 1574, 1541, 1472, 1450, 1368, 1309, 1293, 1256, 1245, 1216, 1098, 1077, 1054, 1011, 933, 819, 767, 751, 696, 639, 607, 529 cm^{-1}. ^{1}H NMR (400.13 MHz, DMSO-d_6): δ 11.64 (s, 1H, NH), 7.82-7.80 (m, 2H, *o*-Ph), 7.67-7.65 (m, 2H, *o*-Ph), 7.58-7.53 (m, 3H, *m,p*-Ph), 7.47-7.44 (m, 2H, *m*-Ph), 7.23 (s, 1H, CN-Ph), 7.11-7.10 (m, 1H, H-3, pyrrole), 6.78-6.77 (m, 1H, H-4, pyrrole), 6.63 (s, 2H, NH_2). ^{13}C NMR (100.6 MHz, DMSO-d_6): δ 154.6, 149.4, 139.1, 137.8, 134.7, 131.3, 130.4, 129.3, 128.9, 128.6 (2C), 128.6 (2C), 128.5 (2C), 126.4 (2C), 117.0, 116.3, 115.6, 113.7, 108.9, 91.8, 89.7.

*3-Amino-5-(1-vinyl-4,5,6,7-tetrahydro-1H-indol-2-yl)-[1,1'-biphenyl]-2,4-dicarbonitrile **(3f)***

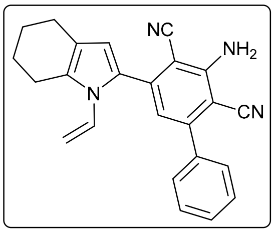

Yield 321 mg (88%), yellow crystals 182-184°C; R_f = 0.65. [Found: C, 79.21; H, 5.71; N, 15.55%, $C_{24}H_{20}N_4$ requires C, 79.10; H, 5.53; N, 15.37%]; ν_{max} (KBr) 3471, 3356, 3240, 2927, 2853, 2214, 2161, 1640, 1585, 1572, 1550, 1498, 1463, 1419, 1380, 1289, 1143, 1076, 1032, 959, 909, 733, 700, 633, 496 cm^{-1}. ^{1}H NMR (400.13 MHz, $CDCl_3$): δ 7.55-7.52 (m, 2H, Ph), 7.50-7.45 (m, 3H, Ph), 6.82 (s, 1H, CN-Ph), 6.73 (dd, J = 15.9, 8.9 Hz, 1H, H_x), 6.51 (s, 1H, pyrrole), 5.31 (br.s, 2H, NH_2), 4.95 (d, J = 8.9 Hz, 1H, H_b), 4.88 (d, J =15.6 Hz, 1H, $1H_a$), 2.68-2.65 (m, 2H, CH_2-7), 2.56-2.53 (m, 2H, CH_2-4), 1.88-1.83 (m, 2H, CH_2-5), 7.79-7.73 (m, 2H, CH_2-6). ^{13}C NMR (100.6 MHz, $CDCl_3$) δ 153.5, 149.1, 141.2, 137.7, 133.7, 130.9, 129.7, 129.0 (2C), 128.5 (2C), 128.4, 120.5, 120.4, 116.6, 116.4, 114.8, 107.1, 94.2, 93.3, 23.9, 23.3, 23.2, 23.1.

*3-Amino-5-(1-benzyl-4,5,6,7-tetrahydro-1H-indol-2-yl)-[1,1'-biphenyl]-2,4-dicarbonitrile **(3g)***

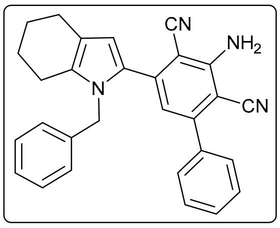

Yield 347 mg (81%), yellow crystals 137-139°C; R_f = 0.67. [Found: C, 81.41; H, 5.81; N, 13.24%, $C_{29}H_{24}N_4$ requires C, 81.28; H, 5.65; N, 13.07%]; ν_{max} (KBr) 3467, 3354, 3239, 3061, 3031, 2928, 2850, 2214, 1632, 1568, 1552, 1496, 1457, 1382, 1287, 1118, 1029, 910, 805, 731, 700, 649, 495 cm^{-1}. ^{1}H NMR (400.13 MHz, $CDCl_3$): δ 7.43-7.28 (m, 7H, Ph), 7.23-7.19 (m, 1H, Ph), 6.87-6.85 (m, 2H, Ph), 6.62 (s, 1H, CN-Ph), 6.51 (s, 1H, pyrrole), 5.29 (s, 2H, NH_2), 5.11 (s, 2H, $\underline{CH_2}$-Ph), 2.61-2.58 (m, 2H, CH_2-7), 2.45-2.43 (m, 2H, CH_2-4), 1.83-1.73 (m, 4H, CH_2-5,6). ^{13}C NMR (100.6 MHz, $CDCl_3$) δ 153.6, 149.1, 141.3, 138.5, 137.4, 134.2, 129.6, 129.0 (2C), 128.8 (2C), 128.6, 128.3 (2C), 127.3, 125.6 (2C), 119.6, 119.5, 116.6, 116.4, 112.8, 94.6, 93.1, 48.0, 23.5, 23.2, 23.1, 22.5.

*2-Amino-4-(1-benzyl-4,5,6,7-tetrahydro-1H-indol-2-yl)-6-(furan-2-yl)isophthalonitrile (**3h**)*

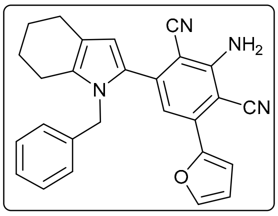

Yield 352 mg (84%), yellow crystals 215-217°C; R_f = 0.66. [Found: C, 77.6; H, 5.45; N, 13.58%, $C_{27}H_{22}N_4O$ requires C, 77.49; H, 5.30; N, 13.39%]; ν_{max} (KBr) 3356, 2923, 2852, 2212, 1723, 1634, 1587, 1558, 1493, 1380, 1294, 1029, 731 cm^{-1}. ^{1}H NMR (400.13 MHz, $CDCl_3$): δ 7.41-7.40 (m, 1H, furan), 7.33-7.29 (m, 2H, Ph), 7.25-7.23 (m, 1H, furan), 7.02-7.01 (m, 1H, Ph), 7.00 (s, 1H, CN-Ph), 6.90-6.88 (m, 2H, Ph), 6.53 (s, 1H, pyrrole), 6.47 (dd, *J* = 3.5, 1.7 Hz, 1H, furan), 5.26 (s, 2H, NH_2), 5.12 (s, 2H, $\underline{CH_2}$-Ph), 2.60 (m, 2H, CH_2-7), 2.46 (m, 2H, CH_2-4), 1.73 (m, 4H, CH_2-5,6). ^{13}C NMR (100.6 MHz, $CDCl_3$) δ 153.8, 149.3, 144.4, 141.2, 138.6, 135.9, 134.3, 129.0 (2C), 128.6, 127.3, 125.7 (2C), 119.6, 116.7 (2C), 115.1, 112.8, 112.6, 112.5, 93.7, 88.2, 48.0, 23.6, 23.2, 23.2, 22.5.

*2-Amino-4-(1-benzyl-4,5,6,7-tetrahydro-1H-indol-2-yl)-6-(thiophen-2-yl)isophthalonitrile (**3i**)*

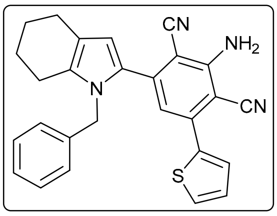

Yield 343 mg (79%), yellow crystals 197-199°C; R_f = 0.66. [Found: C, 74.79; H, 5.27; N, 13.03; S, 7.27%, $C_{27}H_{22}N_4S$ requires C, 74.63; H, 5.10; N, 12.89; S, 7.38%]; ν_{max} (KBr) 3467, 3354, 3239, 2927, 2851, 2211, 2157, 1632, 1566, 1494, 1463, 1437, 1381, 1357, 1289, 1144, 1112, 909, 844, 806, 728 cm^{-1}. ^{1}H NMR (400.13 MHz, $CDCl_3$): δ 7.51-7.45 (m, 1H, thiophene), 7.38-7.37 (m, 1H, thiophene), 7.32-7.21 (m, 3H, Ph, thiophene), 7.08-7.05 (m, 1H, Ph), 6.90-6.88 (m, 2H, Ph), 6.77 (s, 1H, CN-Ph), 6.53 (s, 1H, pyrrole), 5.29 (s, 2H, NH_2), 5.11 (s, 2H, $\underline{CH_2}$-Ph), 2.61-2.58 (m, 2H, CH_2-7), 2.45-2.42 (m, 2H, CH_2-4), 1.83-1.73 (m, 4H, CH_2-5,6). ^{13}C NMR (100.6 MHz, $CDCl_3$) δ 154.0, 141.2, 140.8, 139.0, 138.4, 134.3, 129.1 (2C), 128.6, 128.5, 128.4, 128.2, 127.4, 125.6 (2C), 119.7, 118.3, 116.7, 116.6, 112.9, 94.2, 91.21 48.0, 23.5, 23.2, 23.1, 22.5.

*3-Amino-5-(1-methyl-5-phenyl-1H-pyrrol-2-yl)-[1,1'-biphenyl]-2,4-dicarbonitrile (**3j**)*

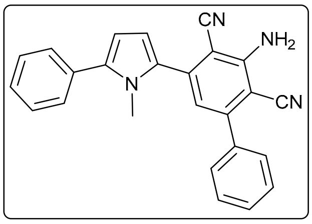

Yield 326 mg (87%), yellow crystals 228-230°C, R_f = 0.68. [Found: C, 80.33; H, 5.01; N, 15.08%, $C_{25}H_{18}N_4$, requires C, 80.19; H, 4.85; N, 14.96%]; ν_{max}(KBr) 3357, 2923, 2854, 2214, 1634, 1576, 1549, 1460, 1285, 758, 728, 700 cm^{-1}. ^{1}H NMR (400.13 MHz, $CDCl_3$): δ 7.60-7.58 (m, 2H, Ph), 7.54-7.42 (m, 7H, Ph), 7.38-7.34 (m, 1H, Ph), 6.88 (s, 1H, CN-Ph), 6.69 (d, *J* = 3.8 Hz, 1H, pyrrole), 6.38 (d, *J* = 3.8 Hz, 1H, pyrrole), 5.39 (s, 2H, NH_2), 3.62 (s, 3H, CH_3). ^{13}C NMR (100.6 MHz, $CDCl_3$) δ 153.5, 149.7, 141.1, 140.5, 137.6, 132.6, 131.9, 129.8, 129.2 (2C), 129.1 (2C), 128.7 (2C), 128.5 (2C), 127.8, 120.1, 116.6, 116.3, 113.6, 109.9, 94.8, 94.0, 34.9.

*3-Amino-5-(1-benzyl-5-phenyl-1H-pyrrol-2-yl)-[1,1'-biphenyl]-2,4-dicarbonitrile (**3k**)*

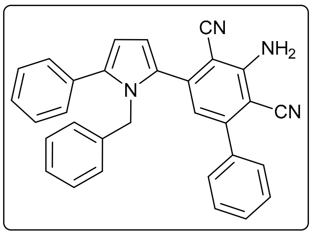

Yield 369 mg (82%); yellow crystals 150-152°C; R_f = 0.68. [Found: C, 82.82; H, 5.02; N, 12.57%, $C_{31}H_{22}N_4$ requires C, 82.64; H, 4.92; N, 12.44%]; ν_{max} (KBr) 3464, 3356, 3062, 3031, 2928, 2216, 1721, 1630, 1580, 1550, 1500, 1456, 1389, 1352, 1286, 1181, 1075, 1032, 910, 760, 732, 701 cm^{-1}. ^1H NMR (400.13 MHz, $CDCl_3$): δ 7.47-7.45 (m, 3H, Ph), 7.42-7.33 (m, 7H, Ph), 7.14-7.12 (m, 3H, Ph), 6.77 (s, 1H, CN-Ph), 6.67 (d, *J* = 3.7 Hz, 1H, pyrrole), 6.63-6.61 (m, 2H, Ph), 6.44 (d, *J* = 3.7 Hz, 1H, pyrrole), 5.30 (s, 2H, NH_2), 5.24 (s, 2H, $\underline{CH_2}$-Ph). ^{13}C NMR (100.6 MHz, $CDCl_3$) δ 153.2, 149.5, 141.5, 140.5, 138.5, 137.4, 132.8, 131.7, 129.7, 129.3 (2C), 129.0 (2C), 128.9, 128.7 (2C), 128.6 (2C), 128.4 (2C), 127.9, 127.3, 126.0 (2C), 120.2, 116.2, 114.3, 110.7, 95.6, 94.1, 49.8.

*2-Amino-4-(1-benzyl-5-phenyl-1H-pyrrol-2-yl)-6-(furan-2-yl)isophthalonitrile (**3l**)*

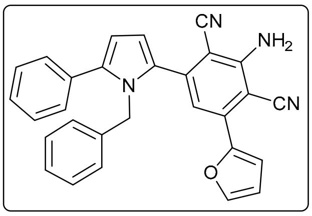

Yield 374 mg (85%), yellow crystals 176-178°C; R_f = 0.67. [Found: C, 79.24; H, 4.75; N, 12.56%, $C_{29}H_{20}N_4O$ requires C, 79.07; H, 4.58; N, 12.72%]; ν_{max} (KBr) 3357, 2922, 2853, 2213, 1633, 1586, 1554, 1479, 1454, 1294, 1029, 910, 755, 732, 701 cm^{-1}. ^1H NMR (400.13 MHz, $CDCl_3$): δ 7.56-7.54 (m, 1H, furan), 7.44-7.32 (m, 5H, Ph), 7.27-7.26 (m, 1H, Ph), 7.21 (s, 1H, CN-Ph), 7.14-7.10 (m, 3H, Ph, furan), 6.70 (d, *J* = 3.7 Hz, 1H, pyrrole), 6.64-6.62 (m, 2H, Ph), 6.57 (dd, *J* = 3.4, 1.6 Hz, 1H, furan), 6.45 (d, *J* = 3.7 Hz, 1H, pyrrole), 5.30-5.23 (m, 4H, NH_2, $\underline{CH_2}$-Ph). ^{13}C NMR (100.6 MHz, $CDCl_3$) δ 153.5, 149.2, 144.6, 141.4, 140.5, 138.5, 136.1, 132.9, 131.7, 129.4 (2C), 128.7 (2C), 128.6 (2C), 127.9, 127.3, 126.0 (2C), 116.6, 116.2, 115.7, 114.3, 112.9, 112.8, 110.8, 94.7, 89.0, 49.7.

*2-Amino-4-(1-benzyl-5-phenyl-1H-pyrrol-2-yl)-6-(thiophen-2-yl)isophthalonitrile (**3m**)*

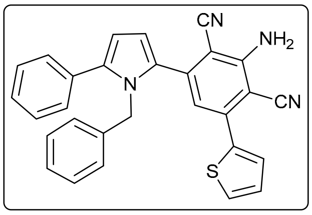

Yield 402 mg (88%), yellow crystals 203-205°C; R_f = 0.66. [Found: 76.01; H, 4.32; N, 12.04; S, 6.84%, $C_{29}H_{20}N_4S$ requires C, 76.29; H, 4.42; N, 12.27; S, 7.02%]; ν_{max} (KBr) 3355, 2921, 2852, 2213, 1721, 1632, 1576, 1546, 1458, 1425, 1288, 1254, 910, 845, 759, 731, 702 cm^{-1}. ^1H NMR (400.13 MHz, $CDCl_3$): δ 7.63-7.58 (m, 1H, thiophene), 7.50-7.47 (m, 1H, thiophene), 7.41-7.32 (m, 5H, Ph), 7.18-7.12 (m, 4H, Ph, thiophene), 6.91 (s, 1H, CN-Ph), 6.67-6.65 (m, 1H, pyrrole), 6.62-6.61 (m, 2H, Ph), 6.43-6.41 (m, 1H, pyrrole), 5.28 (s, 2H, NH_2), 5.24 (s, 2H, $\underline{CH_2}$-Ph). ^{13}C NMR (100.6 MHz, $CDCl_3$) δ 153.6, 141.5, 141.2, 140.6, 138.8, 138.4, 132.8, 131.8, 129.4 (2C), 128.7 (2C), 128.7 (4C), 128.5, 127.9, 127.4, 126.0 (2C), 119.3, 116.5, 116.1, 114.3, 110.6, 95.3, 92.2, 49.8.

*(Z)-2-(3-Hydroxy-3-phenyl-1-(1H-pyrrol-2-yl)allylidene)malononitrile (**4a**).*

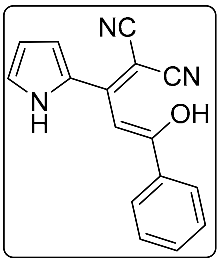

Yield 196 mg (75%), yellow solid, m.p. 175-177 °C; R_f = 0.42. ^{1}H NMR (400.13 MHz, DMSO-d_6): δ 12.08 (br. s, 1H, OH), 8.36 (br. s, 1H, NH), 8.00-7.98 (m, 2H, Ph), 7.59-7.57 (m, 3H, Ph), 7.51-7.50 (m, 1H, H-5, pyrrole), 7.44-7.42 (m, 1H, H-4, pyrrole), 7.31 (s, 1H, =CH), 6.46-6.45 (m, 1H, H-3, pyrrole). ^{13}C NMR (100.6 MHz, DMSO-d_6) δ 158.4, 156.1, 144.8, 131.6, 130.6, 129.0 (2C), 126.4, 125.8 (2C), 125.0, 117.6, 116.2, 112.0, 97.6, 85.6. IR (KBr, cm^{-1}): 3300, 2216, 1630, 1540, 1500, 1467, 1362, 1325, 1052, 994, 910, 780, 699, 642. Elemental analysis calcd (%) for $C_{16}H_{11}N_3O$: C, 73.55; H, 4.24; N, 16.08; found: C, 73.21; H, 4.42; N, 15.88.

*(Z)-2-(3-Hydroxy-3-(2-furyl)-1-(1H-pyrrol-2-yl)allylidene)malononitrile (**4b**).*

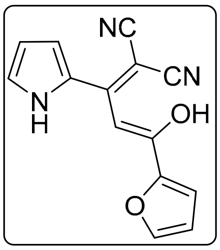

^{1}H NMR (400.13 MHz, DMSO-d_6): δ 12.22 (br. s, 1H, OH), 8.78 (br. s, 1H, NH), 8.06-8.04 (m, 1H, H-5, furan), 7.52-7.51 (m, 1H, H-3, furan), 7.40-7.37 (m, 1H, H-3, pyrrole), 7.16-7.15 (m, 1H, H-4, furan), 7.13 (s, 1H, =CH), 6.81-6.80 (m, 1H, H-5, pyrrole), 6.49-6.46 (m, 1H, H-4, pyrrole).

*(Z)-2-(3-Hydroxy-1-(1H-pyrrol-2-yl)-3-(thiophen-2-yl)allylidene)malononitrile (**4c**).*

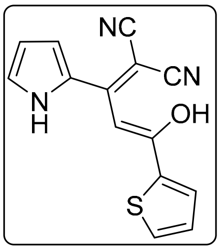

^{1}H NMR (400.13 MHz, DMSO-d_6): δ 12.14 (br. s, 1H, OH), 8.81 (br. s, 1H, NH), 7.96-7.95 (m, 1H, H-5, thiophene), 7.84-7.83 (m, 1H, H-3, thiophene), 7.52-7.51 (m, 1H, H-3, pyrrole), 7.47-7.45 (m, 1H, H-5, pyrrole), 7.32-7.30 (m, 1H, H-4, thiophene), 7.24 (s, 1H, =CH), 6.47-6.46 (m, 1H, H-4, pyrrole). ^{13}C NMR (100.6 MHz, DMSO-d_6) δ 157.0, 154.7, 145.3, 133.9, 132.1, 129.2, 129.0, 127.3, 124.9, 117.3, 116.9, 112.3, 97.0, 83.8.

*(Z)-3-Amino-1-(2-(furan-2-yl)-2-oxoethylidene)-1H-pyrrolizine-2-carbonitrile (**6b**).*

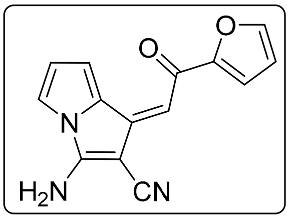

Yield 161 mg (64%), orange solid, m.p. 206-208 °C; R_f = 0.36. ^{1}H NMR (400.13 MHz, DMSO-d_6):δ 9.15 (s, 2H, NH_2), 7.97-7.94 (m, 1H, H-5, furan), 7.53-7.51 (m, 1H, H-5, pyrrole), 7.47-7.44 (m, 1H, H-3, furan), 6.33-6.31 (m, 1H, H-4, furan), 6.72-6.69 (m, 1H, H-3, pyrrole), 6.57 (s, 1H, =CH), 6.48-6.45 (m, 1H, H-4, pyrrole). ^{13}C NMR (100.6 MHz, DMSO-d_6): δ 175.2, 154.9, 154.2, 146.4, 142.0, 131.5, 118.3, 117.1, 116.7, 115.6, 115.5, 112.7, 104.1, 67.2. IR (KBr, cm^{-1}): 3134, 2205, 1687, 1622, 1557, 1540, 1492, 1466, 1398, 1260, 1235, 1206, 1114, 1087, 1067, 1039, 1017, 968, 886, 811, 722, 669. Elemental analysis calcd (%) for $C_{14}H_9N_3O_2$: C, 66.93; H, 3.61; N, 16.73; found: C, 67.12; H, 3.80; N, 16.91.

(*Z*)-3-Amino-1-(2-oxo-2-(thiophen-2-yl)ethylidene)-1*H*-pyrrolizine-2-carbonitrile (6c). Yield 147 mg (55%), red solid, m.p. 283-285 °C; R_f = 0.36. ^{1}H NMR (400.13 MHz, DMSO-d_6): δ 9.15 (s, 2H, NH_2), 7.91-7.90 (m, 1H, H-5, thiophene), 7.88-7.86 (m, 1H, H-3,

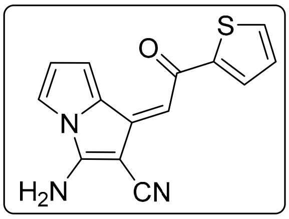

thiophene), 7.53-7.51 (m, 1H, H-5, pyrrole), 7.45-7.44 (m, 1H, H-3, pyrrole), 7.25-7.22 (m, 1H, H-4, thiophene), 6.56 (s, 1H, =CH), 6.48-6.45 (m, 1H, H-4, pyrrole). ^{13}C NMR (100.6 MHz, DMSO-d$_6$): δ 179.1, 155.0, 146.9, 142.0, 133.3, 131.5, 130.4, 128.7, 118.2, 117.2, 116.7, 115.6, 104.3, 67.1. Elemental analysis calcd (%) for $C_{14}H_9N_3OS$: C, 62.91; H, 3.39; N, 15.72; S, 11.99; found: C, 63.05; H, 3.55; N, 15.91; S, 11.86.

Synthesis of 3-amino-1-acylethylidene-2-cyanopyrrolizine (6a)

The triethylamine (101 mg, 1 mmol) was added to the solution of pyrrolyldienol **4a** (1 mmol) in EtOH (40 mL) and refluxed for 30 min. After the reaction mixture was cooled to room temperature, the resulting crystalline precipitate **6a** filtered off on a Schott filter and dried under vacuum.

(*Z*)-3-Amino-1-(2-oxo-2-phenylethylidene)-1*H*-pyrrolizine-2-carbonitrile (6a).

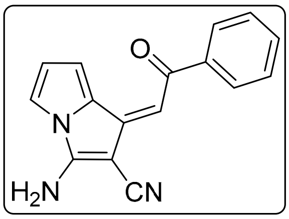

Yield 209 mg (80%), brown solid, m.p. 158-160 °C; R_f = 0.35. ^{1}H NMR (400.13 MHz, DMSO-d$_6$): δ 9.15 (s, 2H, NH_2), 7.97-7.95 (m, 2H, H-5, H-3, pyrrole), 7.61-7.53 (m, 5H, Ph), 6.68 (m, 1H, H-4, pyrrole), 6.46 (s, 1H, =CH). ^{13}C NMR (100.6 MHz, DMSO-d$_6$): δ 186.4, 154.9, 142.3, 139.5, 131.9, 131.6, 128.7 (2C), 127.5 (2C), 118.3, 117.1, 116.7, 115.7, 104.5, 67.4. IR (KBr, cm^{-1}): 3314, 2206, 1638, 1591, 1512, 1443, 1398, 1366, 1282, 1180, 1147, 1057, 966, 872, 823, 757, 698, 608, 531. Elemental analysis calcd (%) for $C_{16}H_{11}N_3O$: C, 73.55; H, 4.24; N, 16.08; found: C, 73.73; H, 4.38; N, 16.27.

(1) Trofimov, B. A.; Sobenina, L. N.; Demenev, A. P.; Mikhaleva, A. b. I., C-Vinylpyrroles as Pyrrole Building Blocks. *Chem. Rev.* **2004,** *104*, 2481-2506.

The NMR spectra:

^{1}H NMR spectrum of 3-amino-5-(1-methyl-1*H*-pyrrol-2-yl)-[1,1'-biphenyl]-2,4-dicarbonitrile (**3a**) in DMSO-d_6.

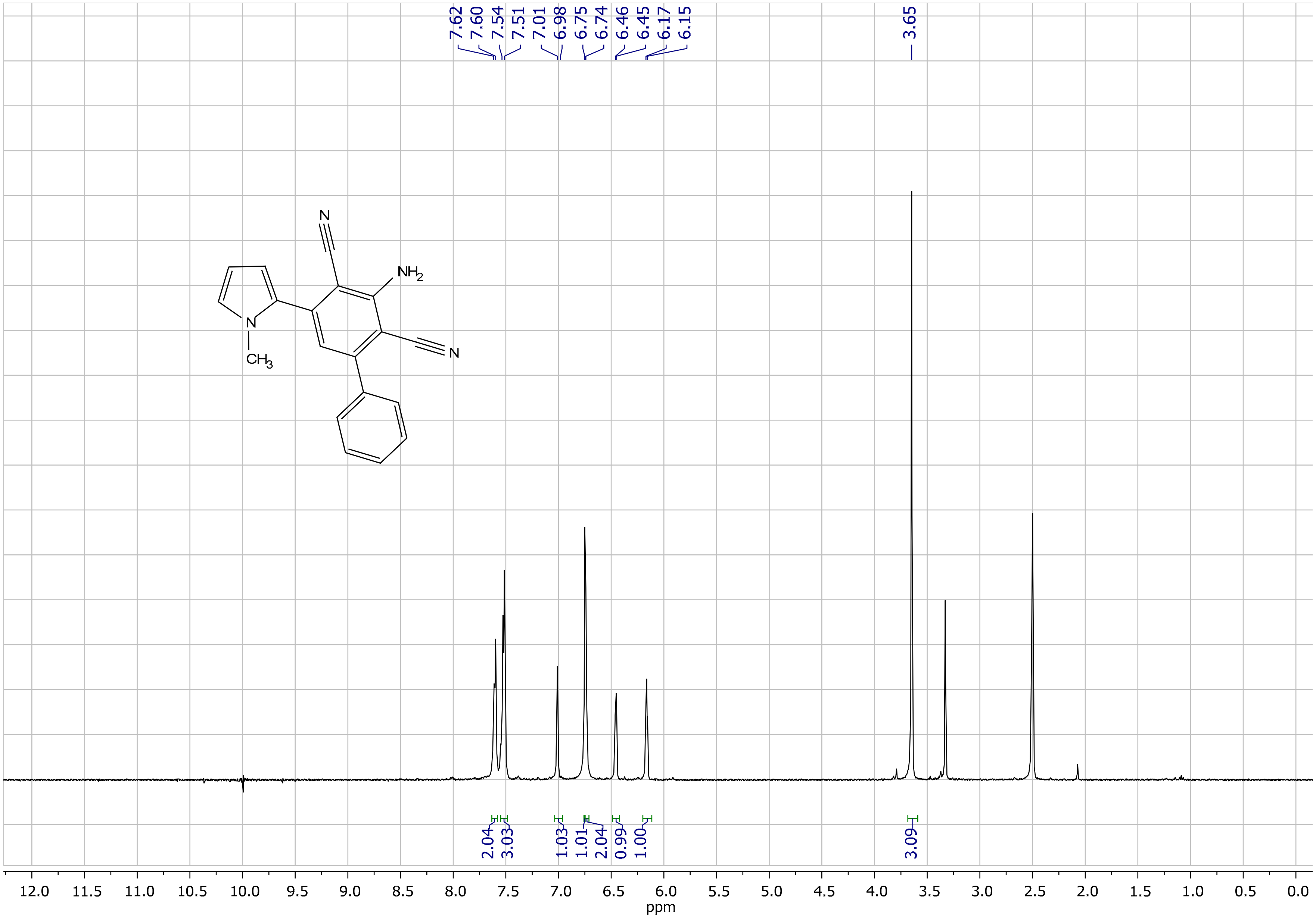

^{13}C NMR spectrum of 3-amino-5-(1-methyl-1*H*-pyrrol-2-yl)-[1,1'-biphenyl]-2,4-dicarbonitrile (**3a**) in DMSO-d$_6$.

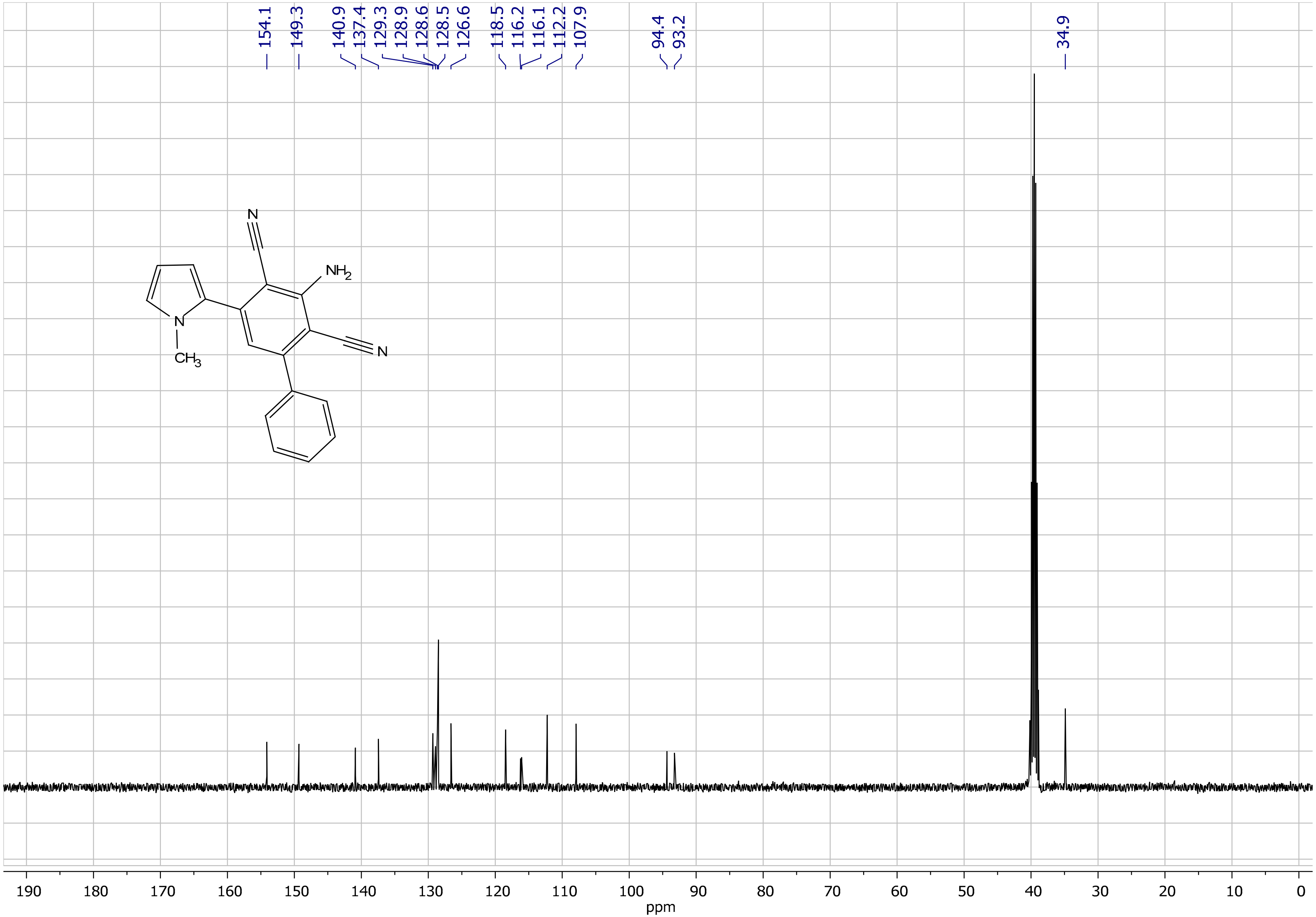

^{1}H NMR spectrum of 3-amino-5-(4-ethyl-5-propyl-1*H*-pyrrol-2-yl)-[1,1'-biphenyl]-2,4-dicarbonitrile (**3b**) in $CDCl_3$.

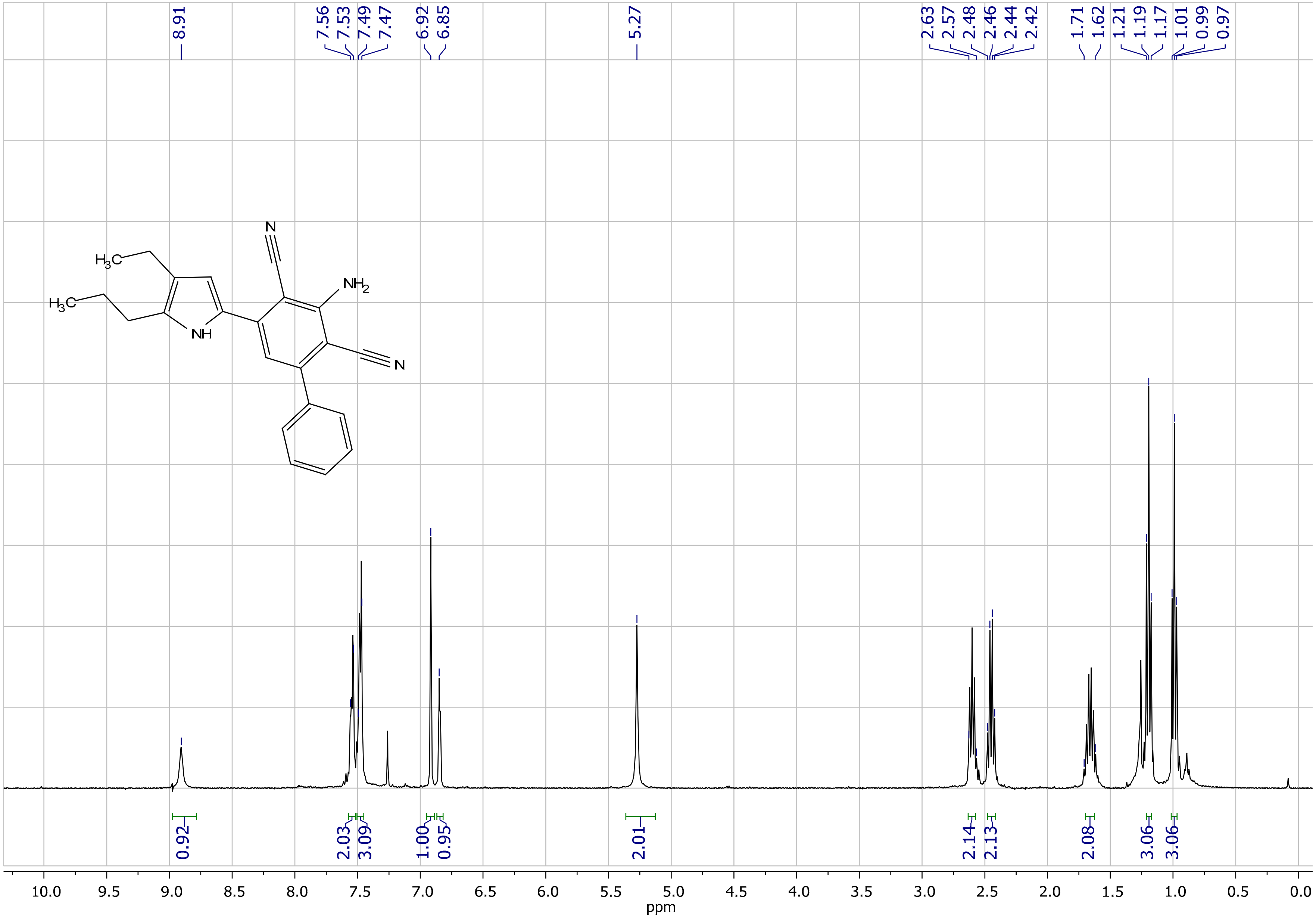

^{13}C NMR spectrum of 3-amino-5-(4-ethyl-5-propyl-1*H*-pyrrol-2-yl)-[1,1'-biphenyl]-2,4-dicarbonitrile (**3b**) in $CDCl_3$.

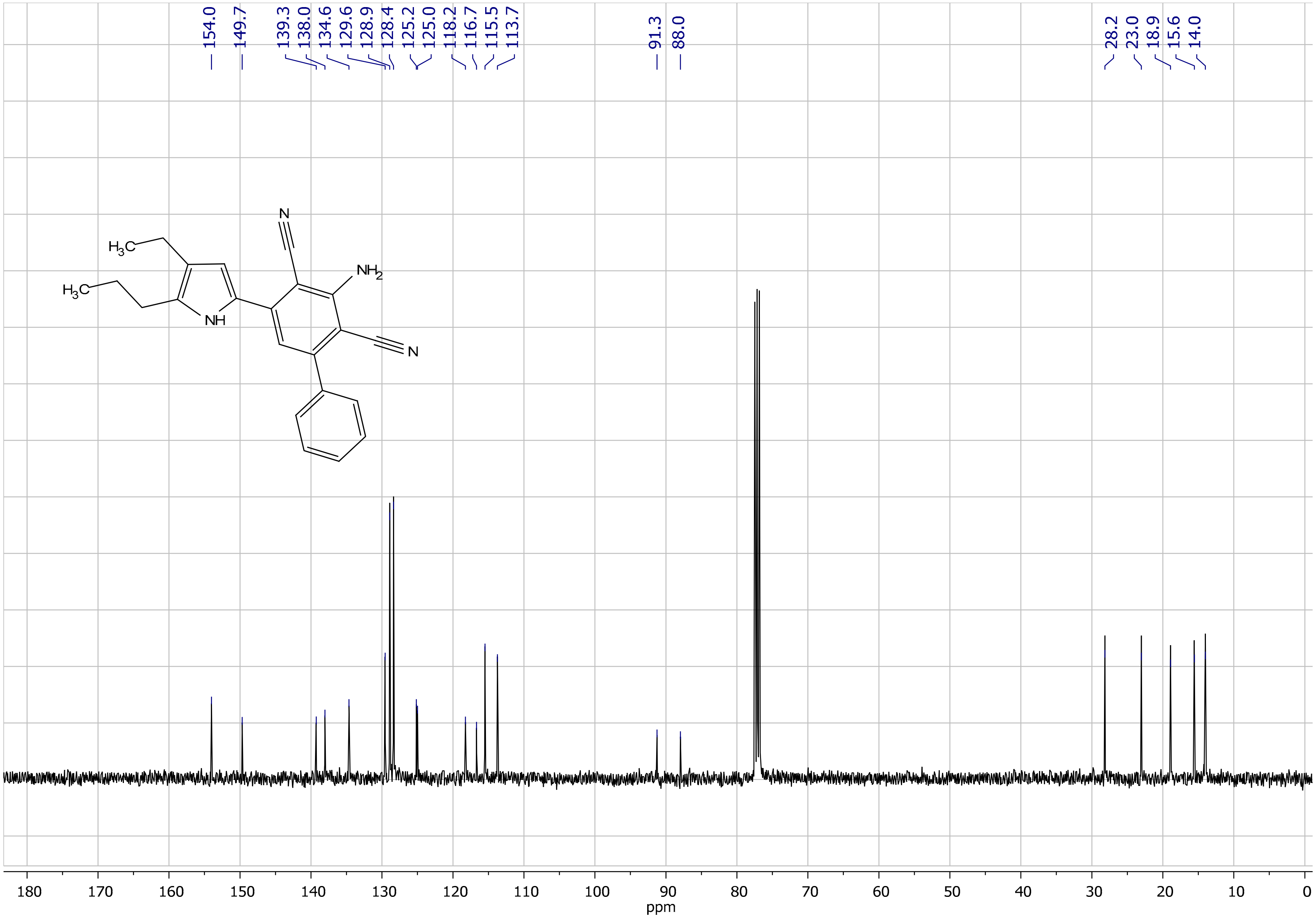

^{1}H NMR spectrum of 3-amino-5-(4,5,6,7-tetrahydro-1*H*-indol-2-yl)-[1,1'-biphenyl]-2,4-dicarbonitrile (**3c**) in DMSO-d$_6$.

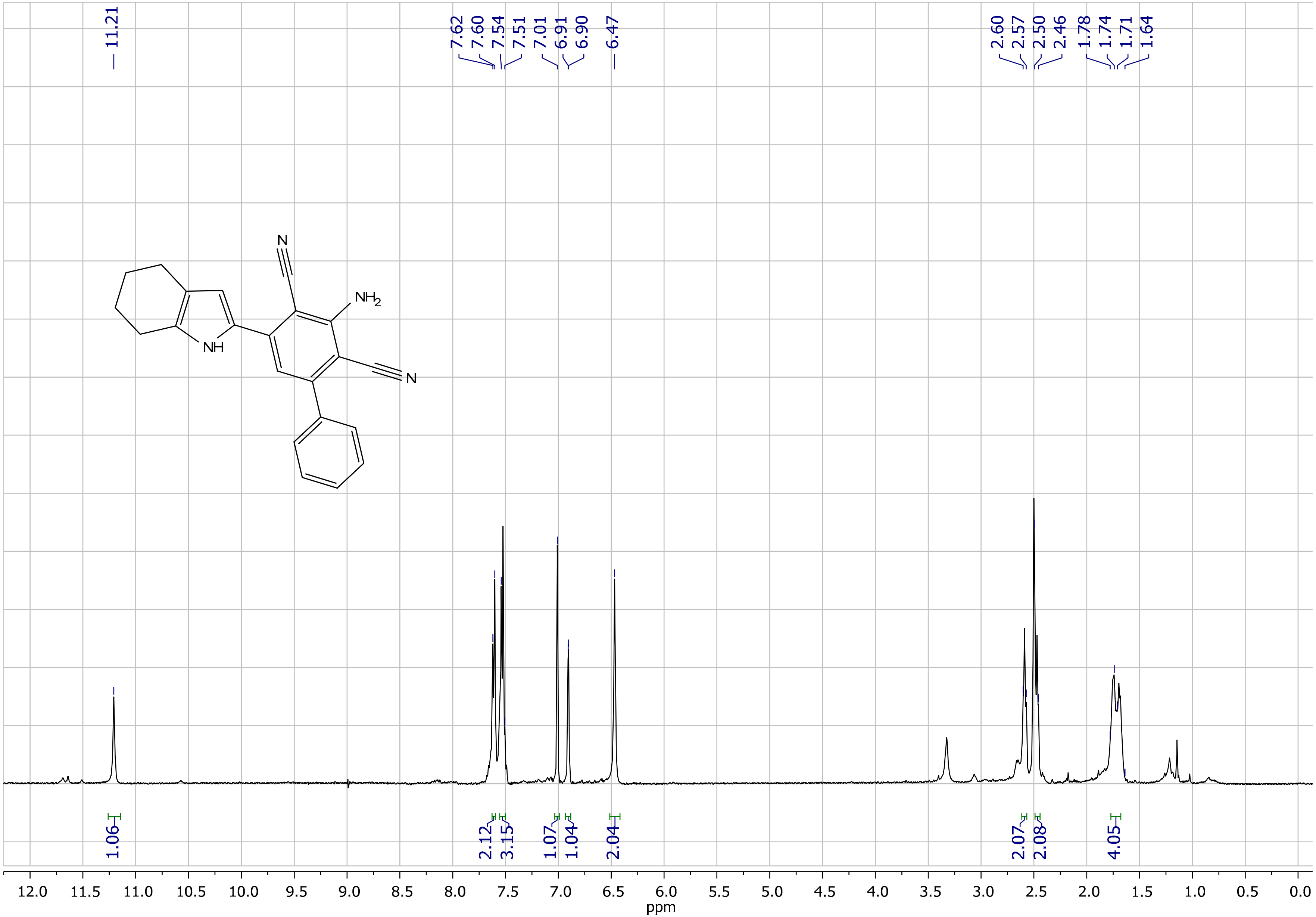

^{13}C NMR spectrum of 3-amino-5-(4,5,6,7-tetrahydro-1*H*-indol-2-yl)-[1,1'-biphenyl]-2,4-dicarbonitrile (**3c**) in DMSO-d_6.

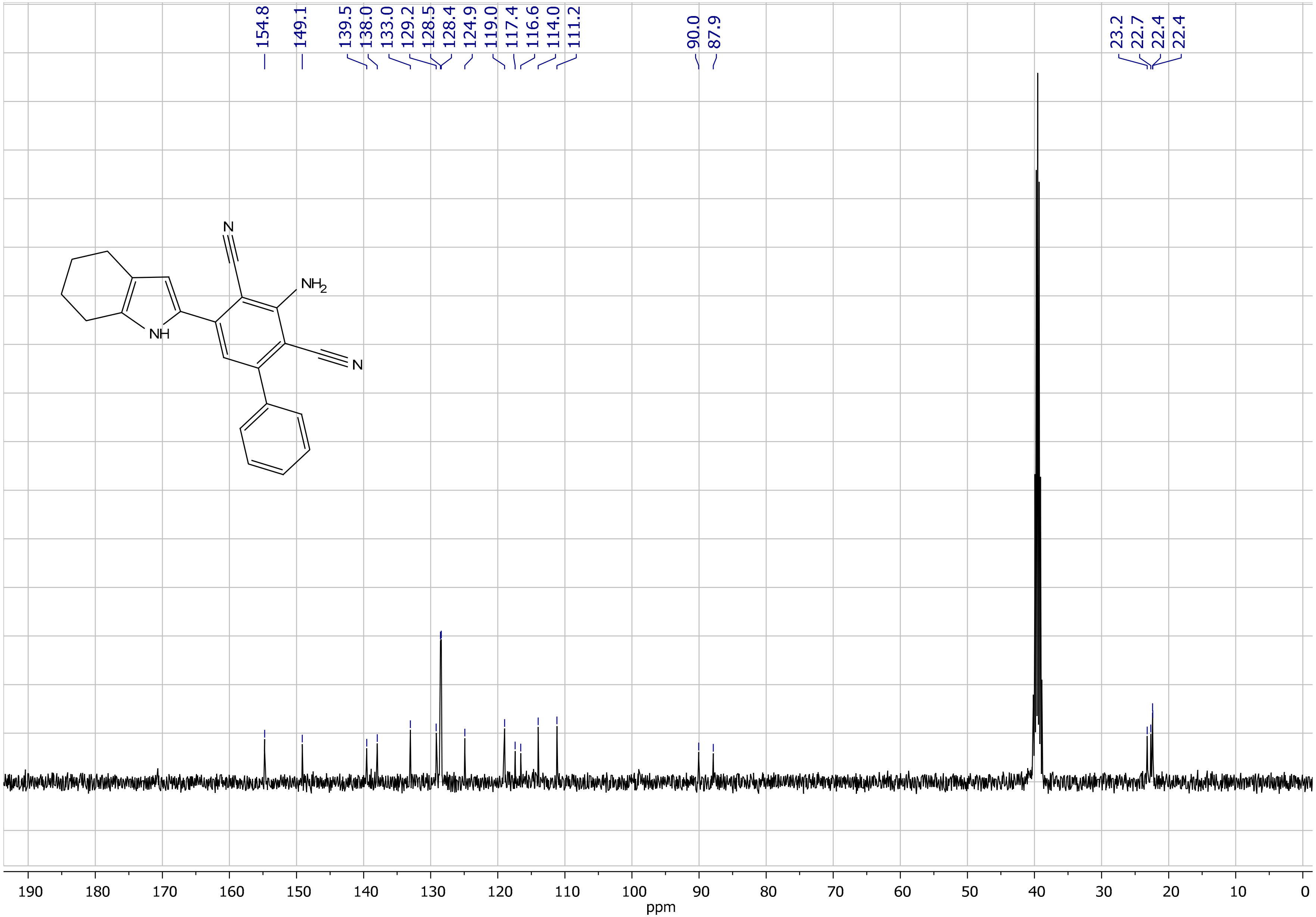

^{1}H NMR spectrum of 3-amino-5-(5-phenyl-1*H*-pyrrol-2-yl)-[1,1'-biphenyl]-2,4-dicarbonitrile (**3d**) in $CDCl_3$.

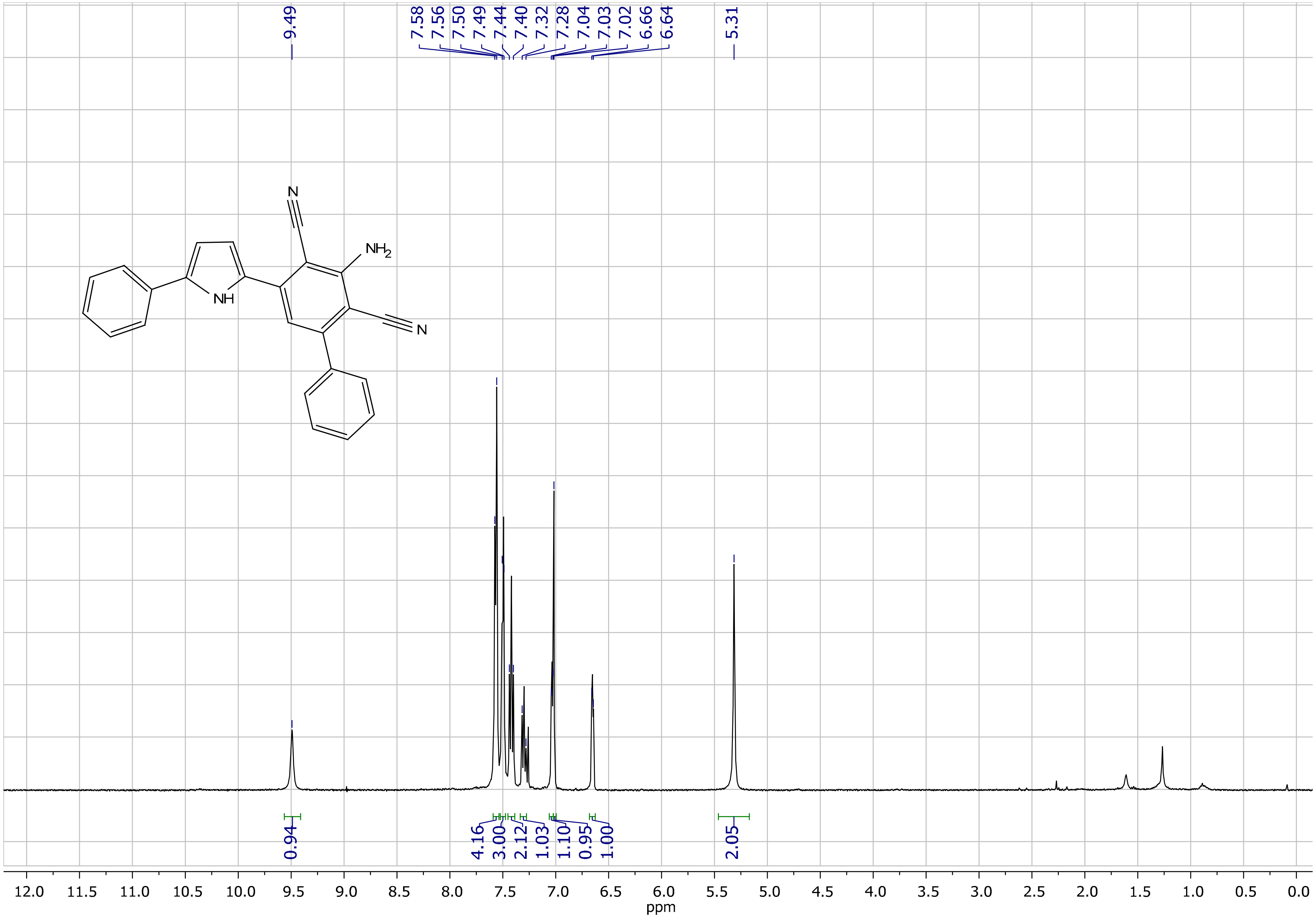

^{13}C NMR spectrum of 3-amino-5-(5-phenyl-1*H*-pyrrol-2-yl)-[1,1'-biphenyl]-2,4-dicarbonitrile (**3d**) in $CDCl_3$.

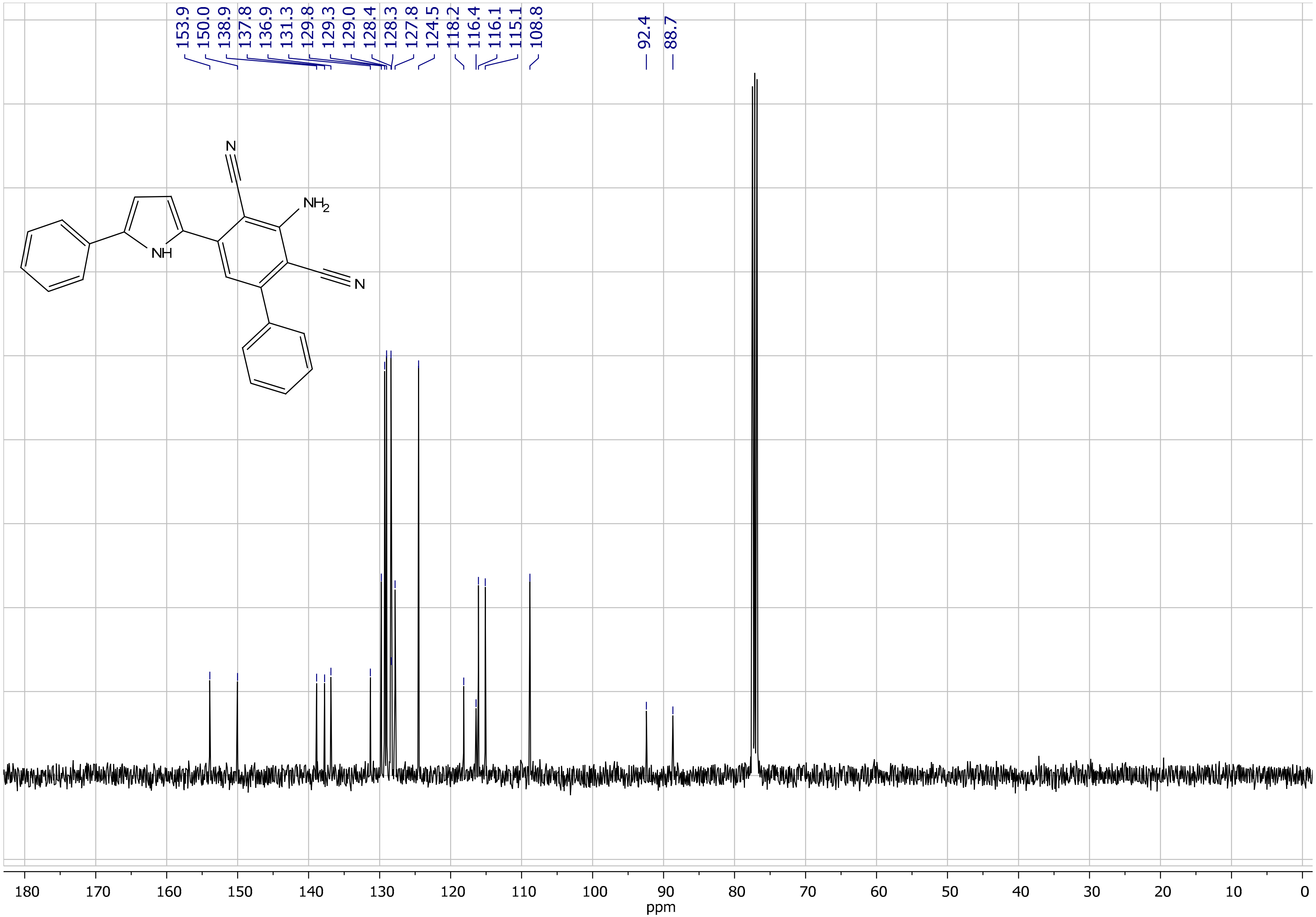

^{1}H NMR spectrum of 3-amino-5-(5-(4-chlorophenyl)-1*H*-pyrrol-2-yl)-[1,1'-biphenyl]-2,4-dicarbonitrile (**3e**) in DMSO-d_6.

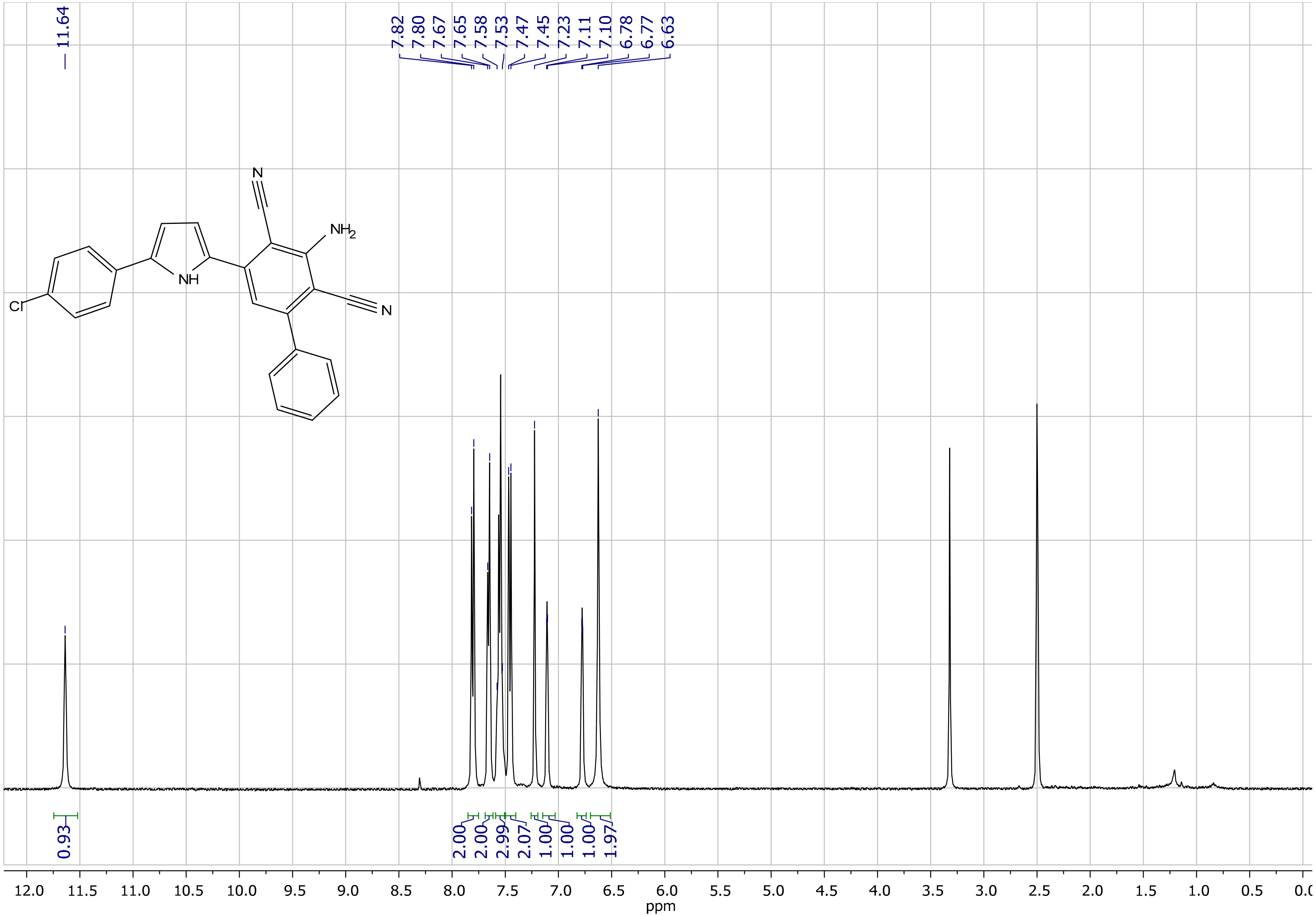

^{13}C NMR spectrum of 3-amino-5-(5-(4-chlorophenyl)-1*H*-pyrrol-2-yl)-[1,1'-biphenyl]-2,4-dicarbonitrile (**3e**) in DMSO-d$_6$.

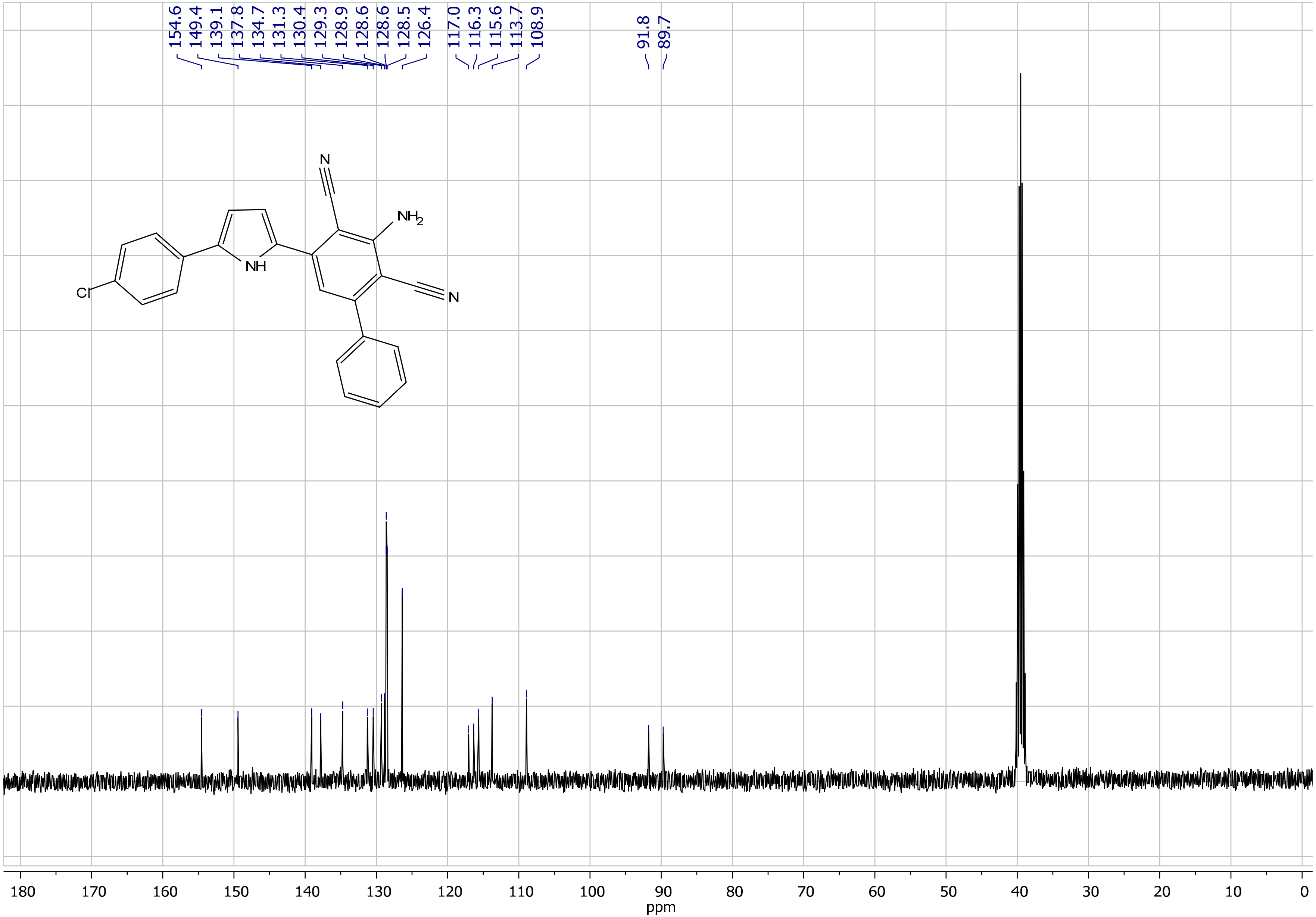

^{1}H NMR spectrum of 3-amino-5-(1-vinyl-4,5,6,7-tetrahydro-1*H*-indol-2-yl)-[1,1'-biphenyl]-2,4-dicarbonitrile (**3f**) in $CDCl_3$.

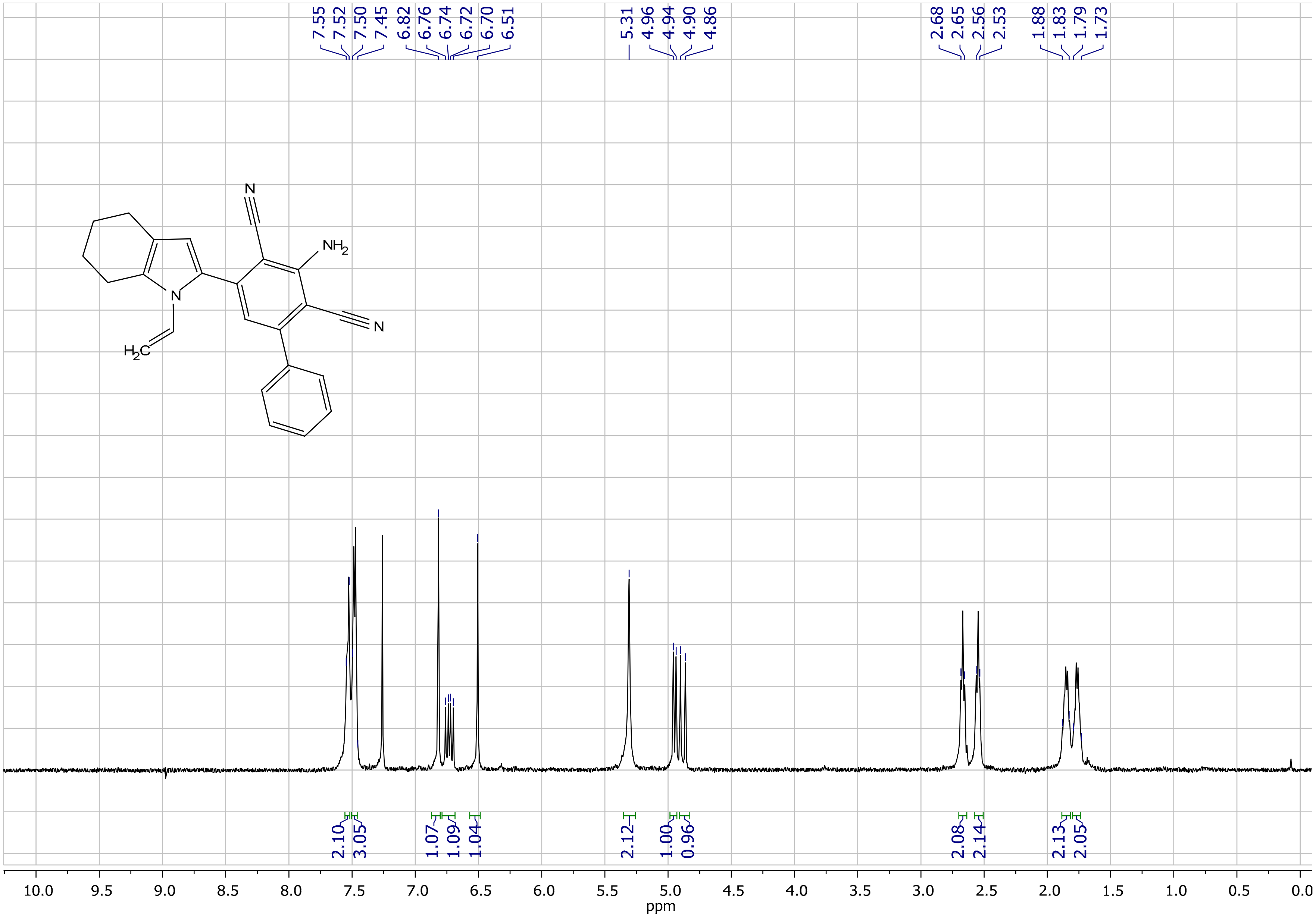

^{13}C NMR spectrum of 3-amino-5-(1-vinyl-4,5,6,7-tetrahydro-1*H*-indol-2-yl)-[1,1'-biphenyl]-2,4-dicarbonitrile (**3f**) in $CDCl_3$.

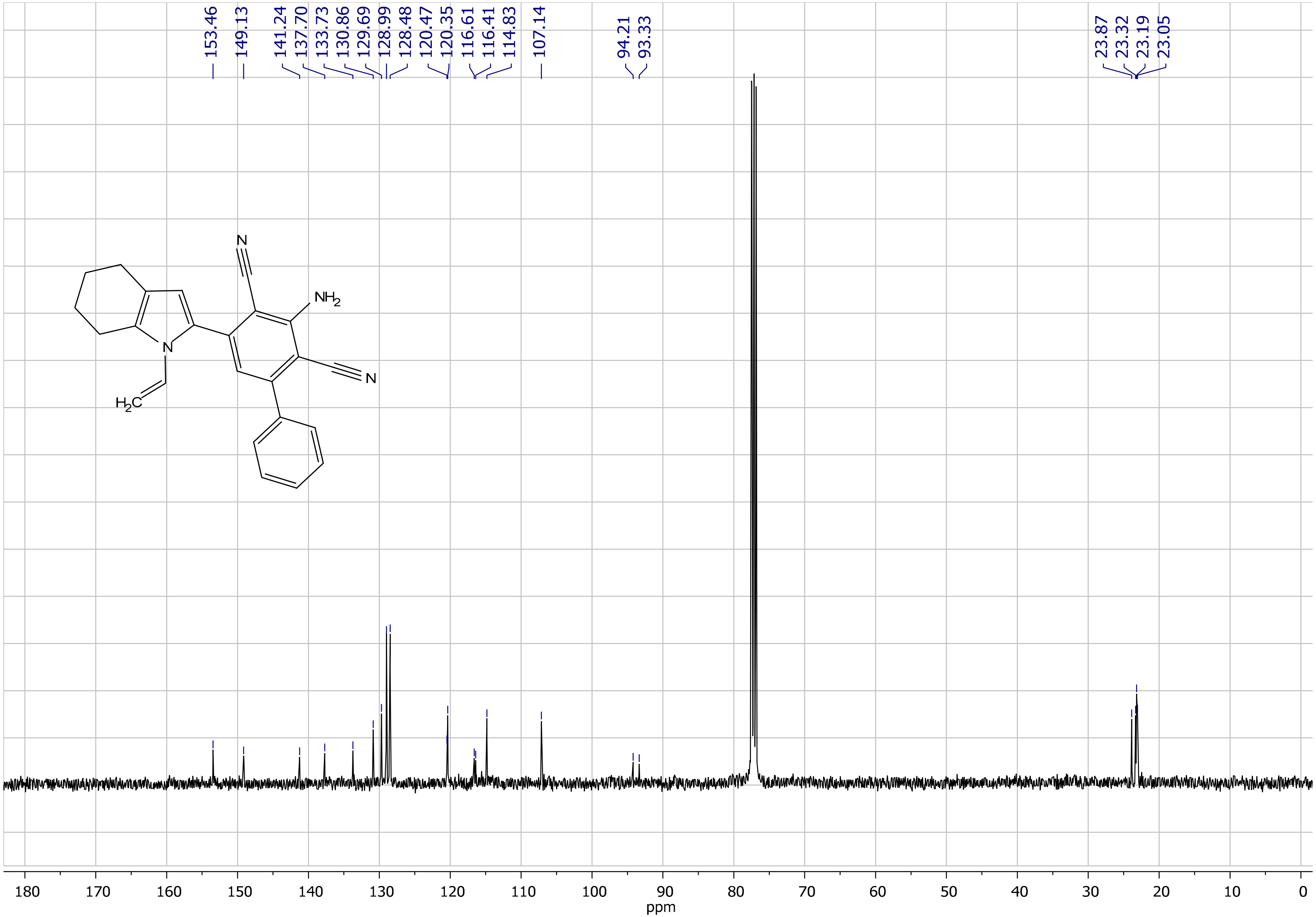

^{1}H NMR spectrum of 3-amino-5-(1-benzyl-4,5,6,7-tetrahydro-1*H*-indol-2-yl)-[1,1'-biphenyl]-2,4-dicarbonitrile (**3g**) in $CDCl_3$.

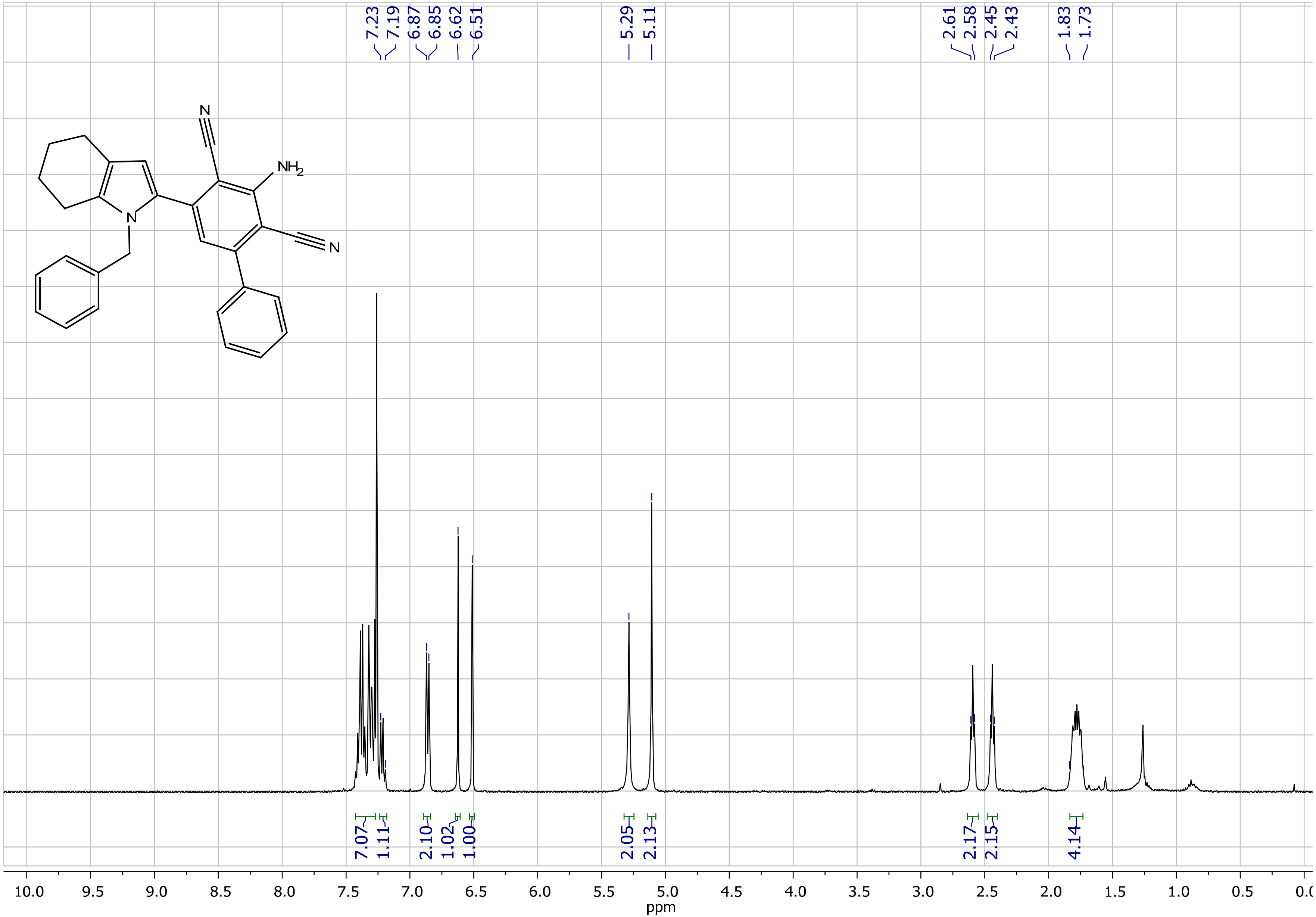

^{13}C NMR spectrum of 3-amino-5-(1-benzyl-4,5,6,7-tetrahydro-1*H*-indol-2-yl)-[1,1'-biphenyl]-2,4-dicarbonitrile (**3g**) in $CDCl_3$.

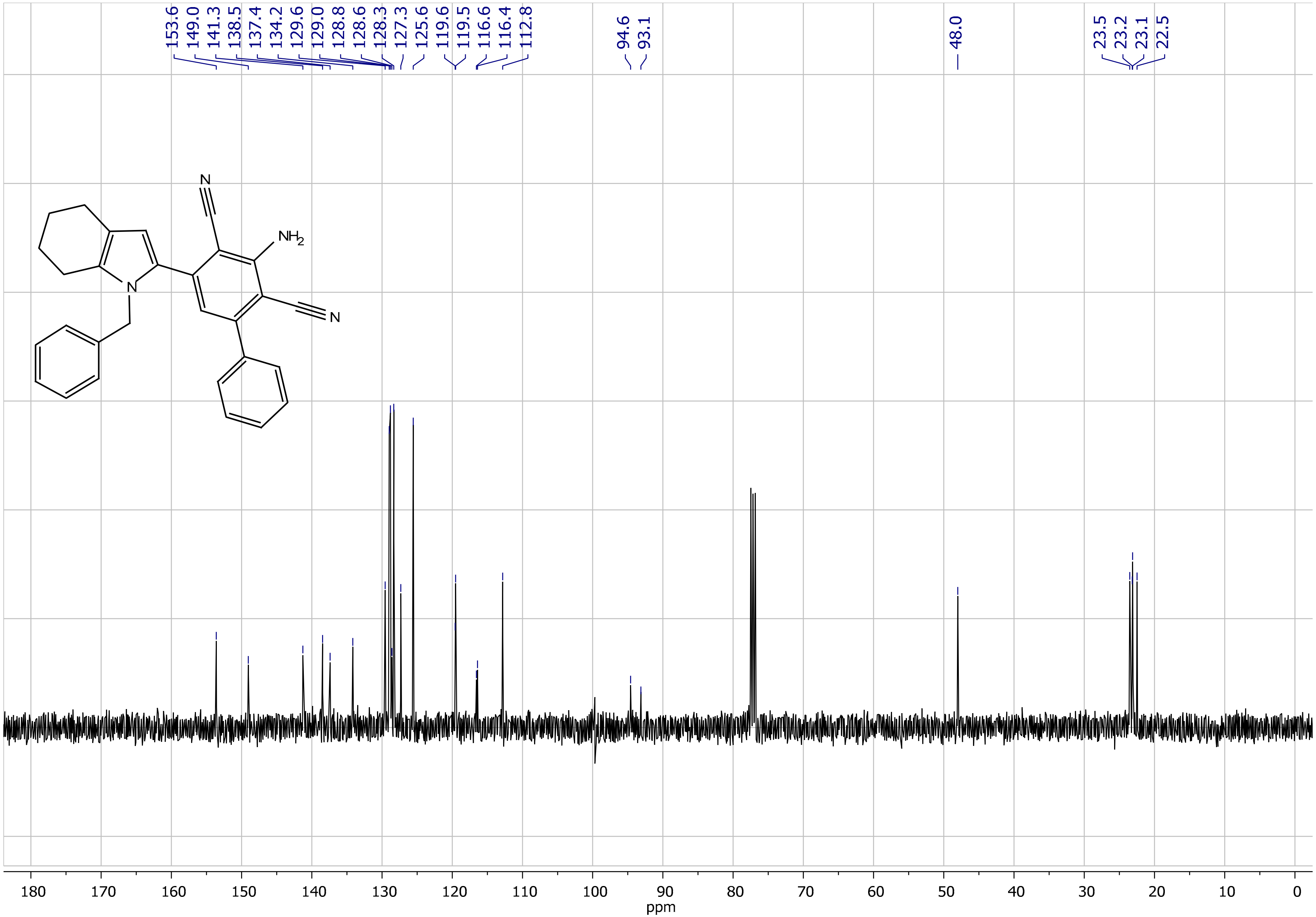

^{1}H NMR spectrum of 2-amino-4-(1-benzyl-4,5,6,7-tetrahydro-1*H*-indol-2-yl)-6-(furan-2-yl)isophthalonitrile (**3h**) in $CDCl_3$.

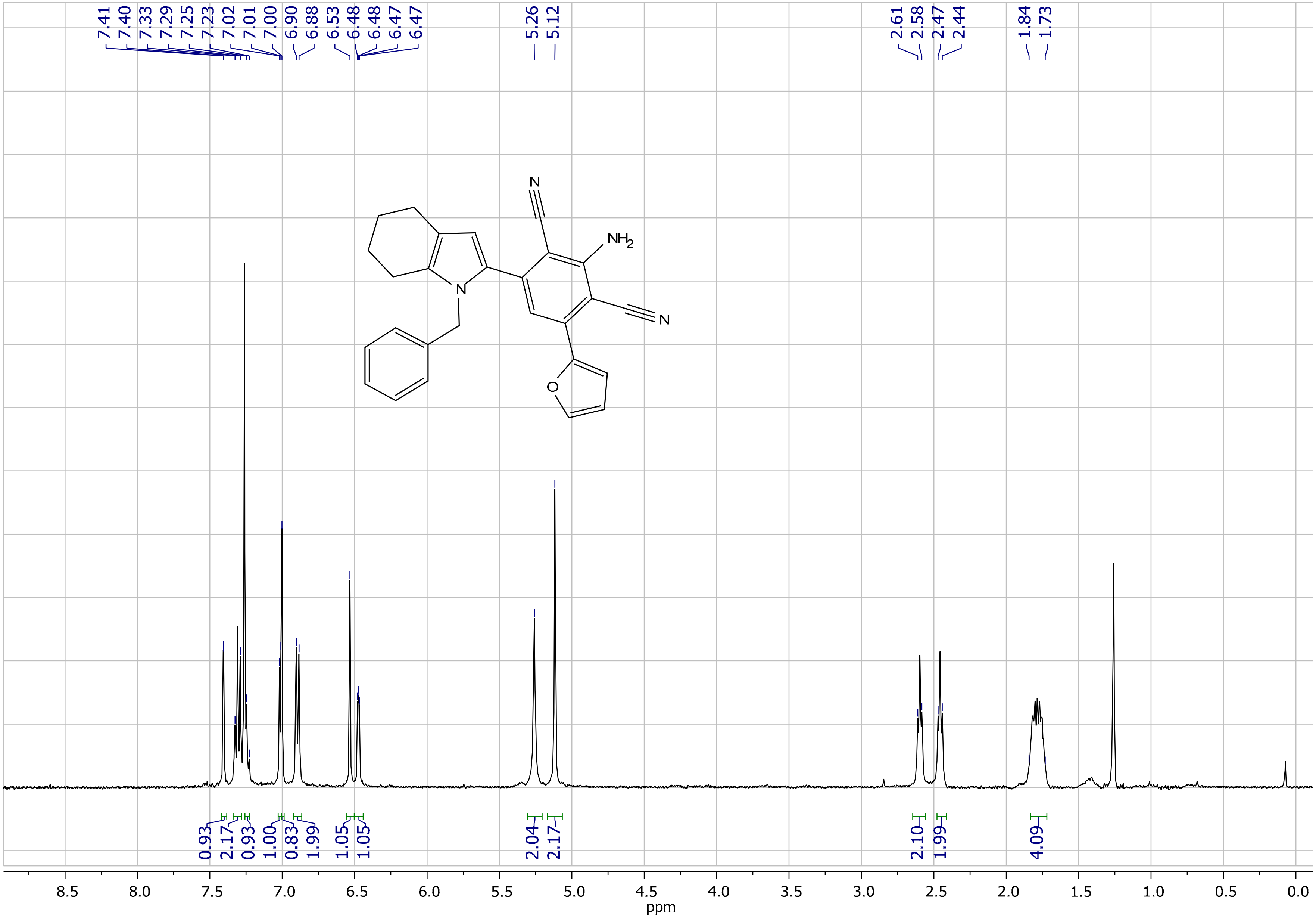

^{13}C NMR spectrum of 2-amino-4-(1-benzyl-4,5,6,7-tetrahydro-1*H*-indol-2-yl)-6-(furan-2-yl)isophthalonitrile (**3h**) in $CDCl_3$.

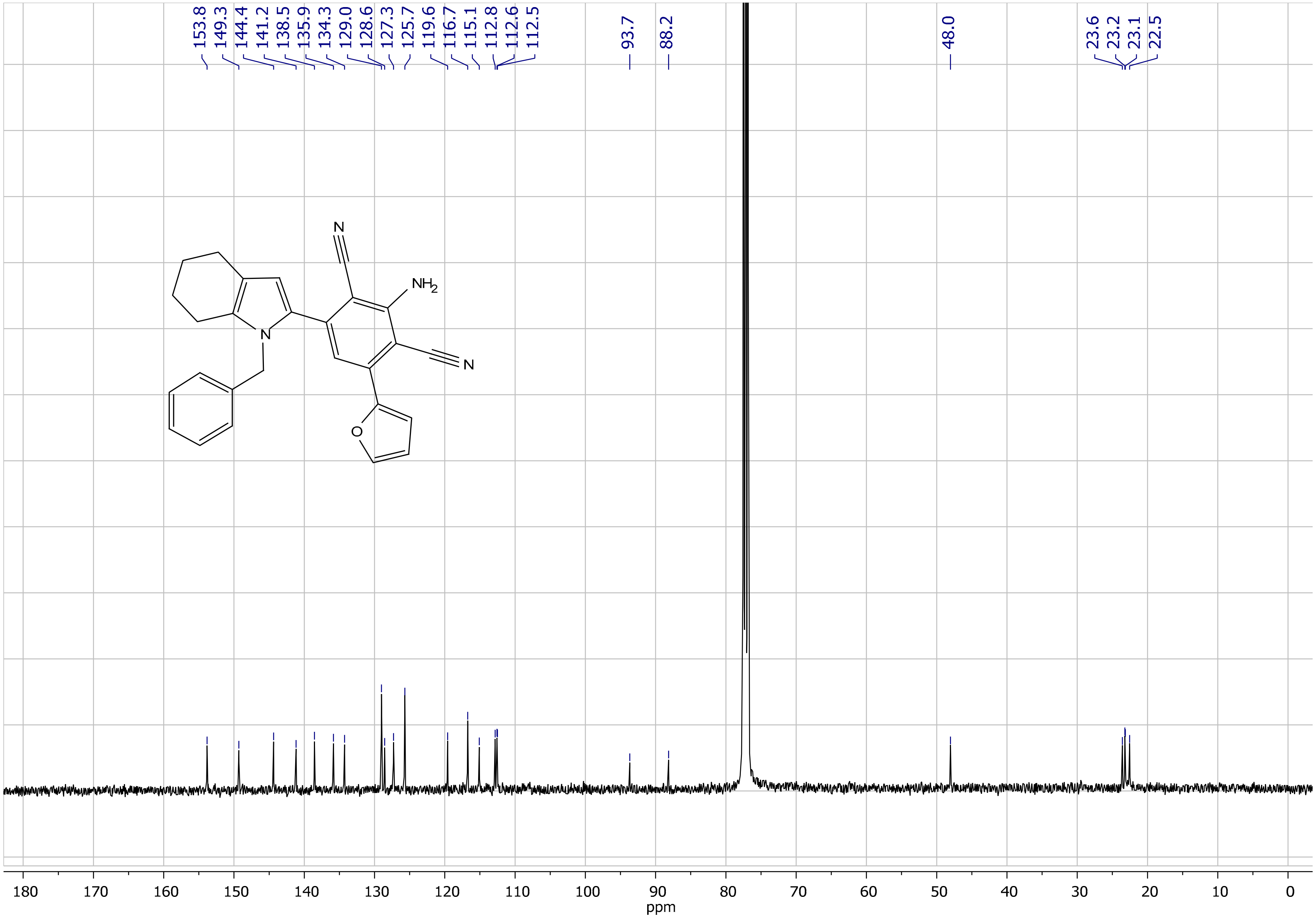

^{1}H NMR spectrum of 2-amino-4-(1-benzyl-4,5,6,7-tetrahydro-1*H*-indol-2-yl)-6-(thiophen-2-yl)isophthalonitrile (**3i**) in $CDCl_3$.

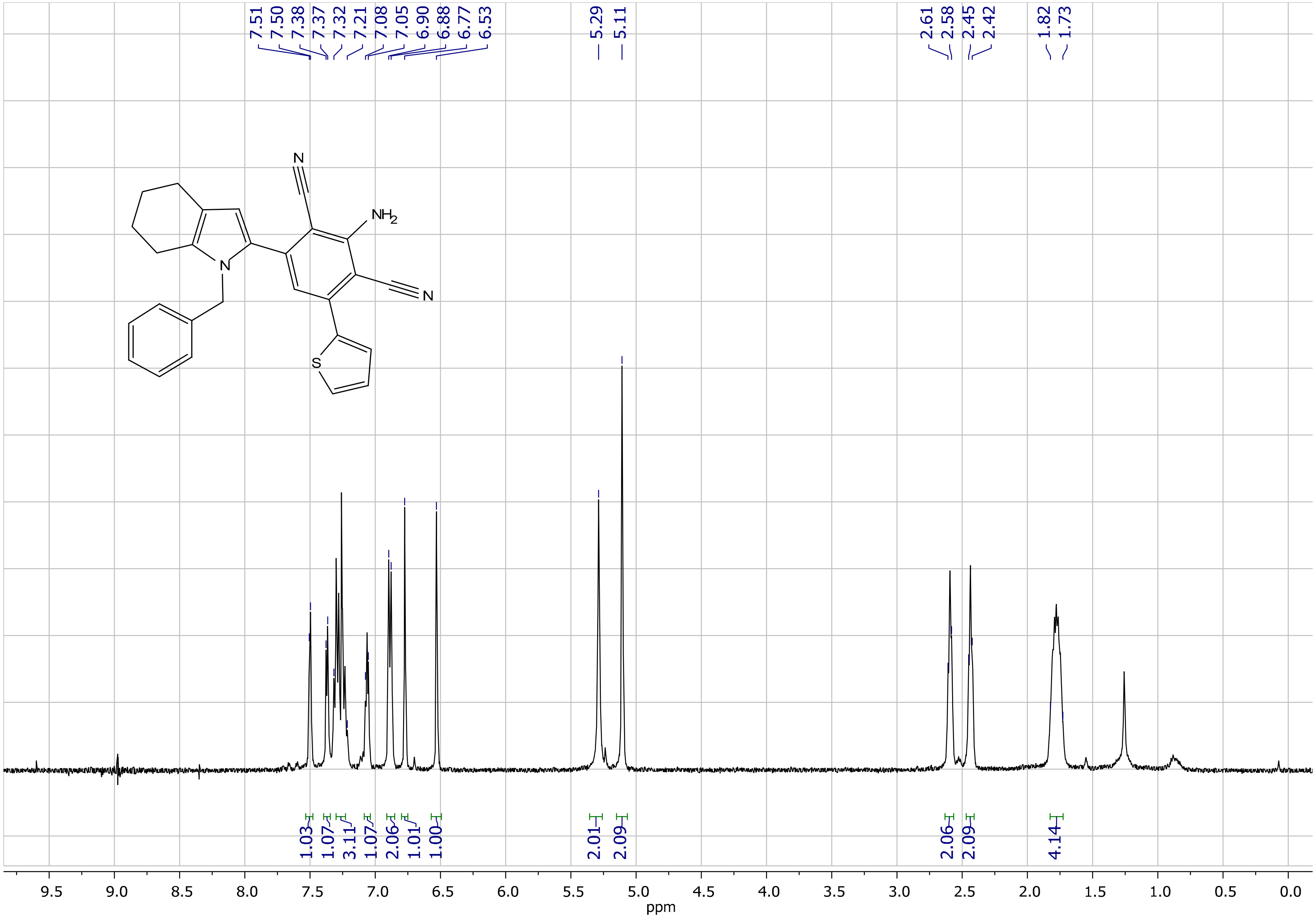

^{13}C NMR spectrum of 2-amino-4-(1-benzyl-4,5,6,7-tetrahydro-1*H*-indol-2-yl)-6-(thiophen-2-yl)isophthalonitrile (**3i**) in $CDCl_3$.

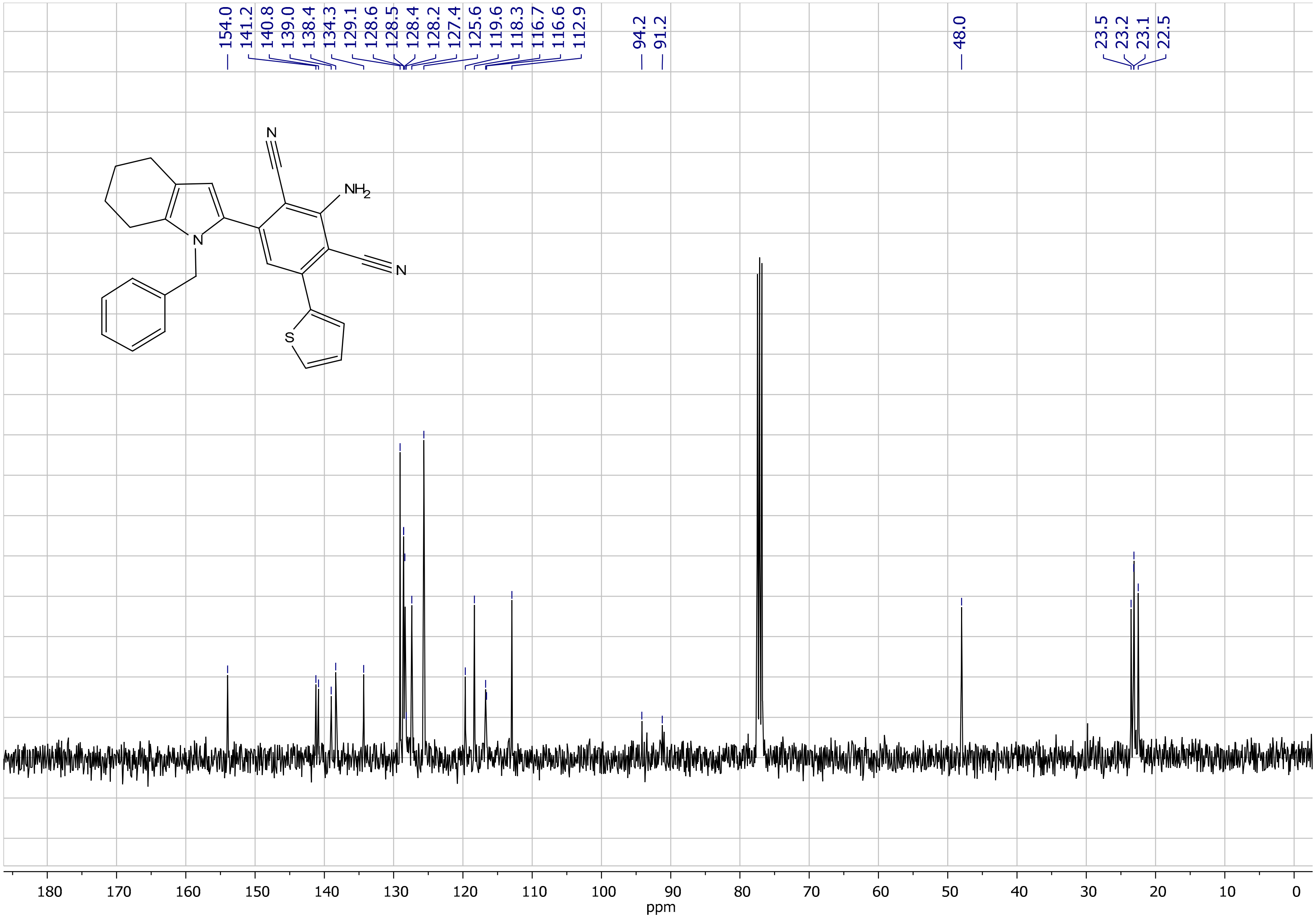

^{1}H NMR spectrum of 3-amino-5-(1-methyl-5-phenyl-1*H*-pyrrol-2-yl)-[1,1'-biphenyl]-2,4-dicarbonitrile (**3j**) in $CDCl_3$.

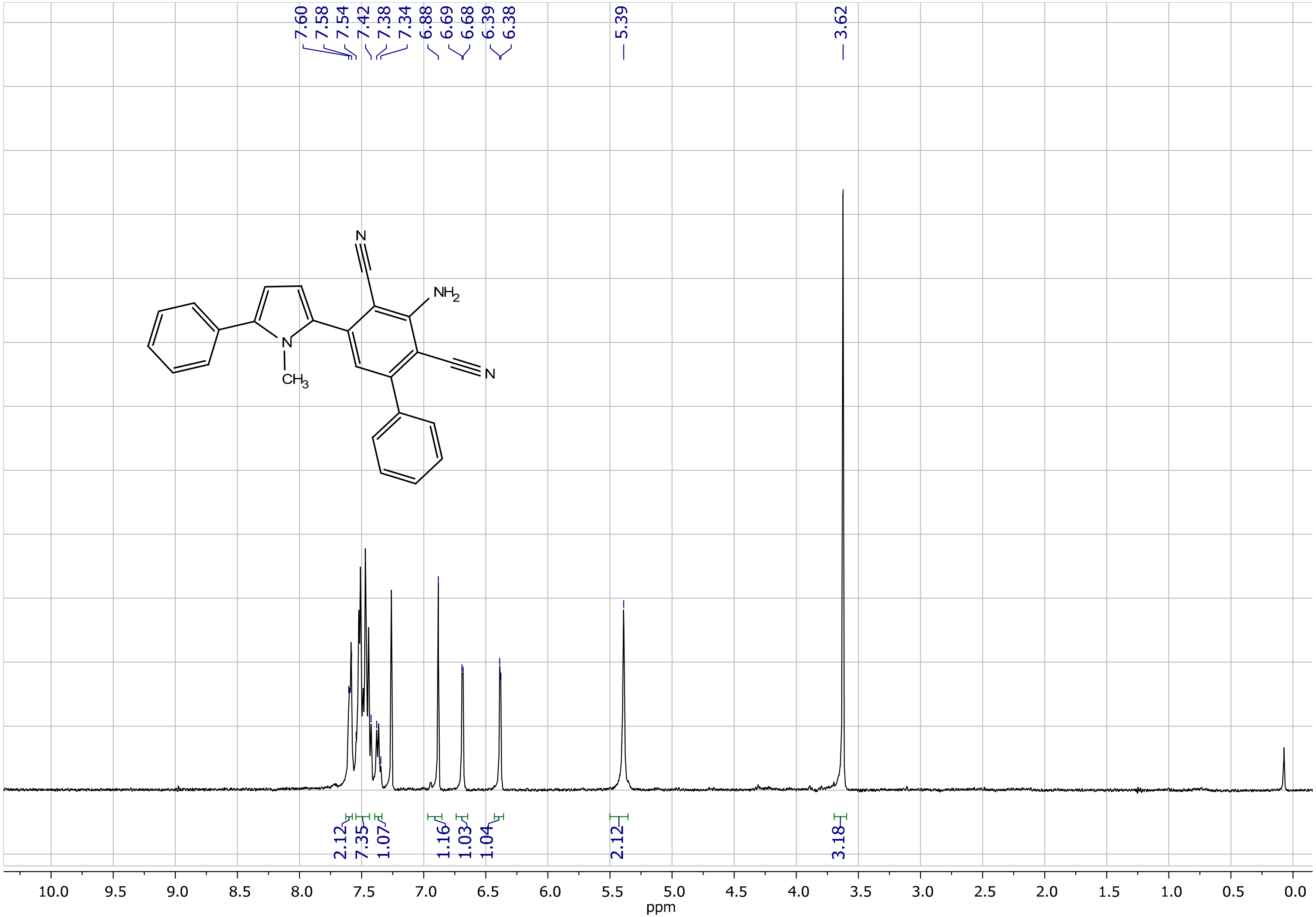

^{13}C NMR spectrum of 3-amino-5-(1-methyl-5-phenyl-1*H*-pyrrol-2-yl)-[1,1'-biphenyl]-2,4-dicarbonitrile (**3j**) in $CDCl_3$.

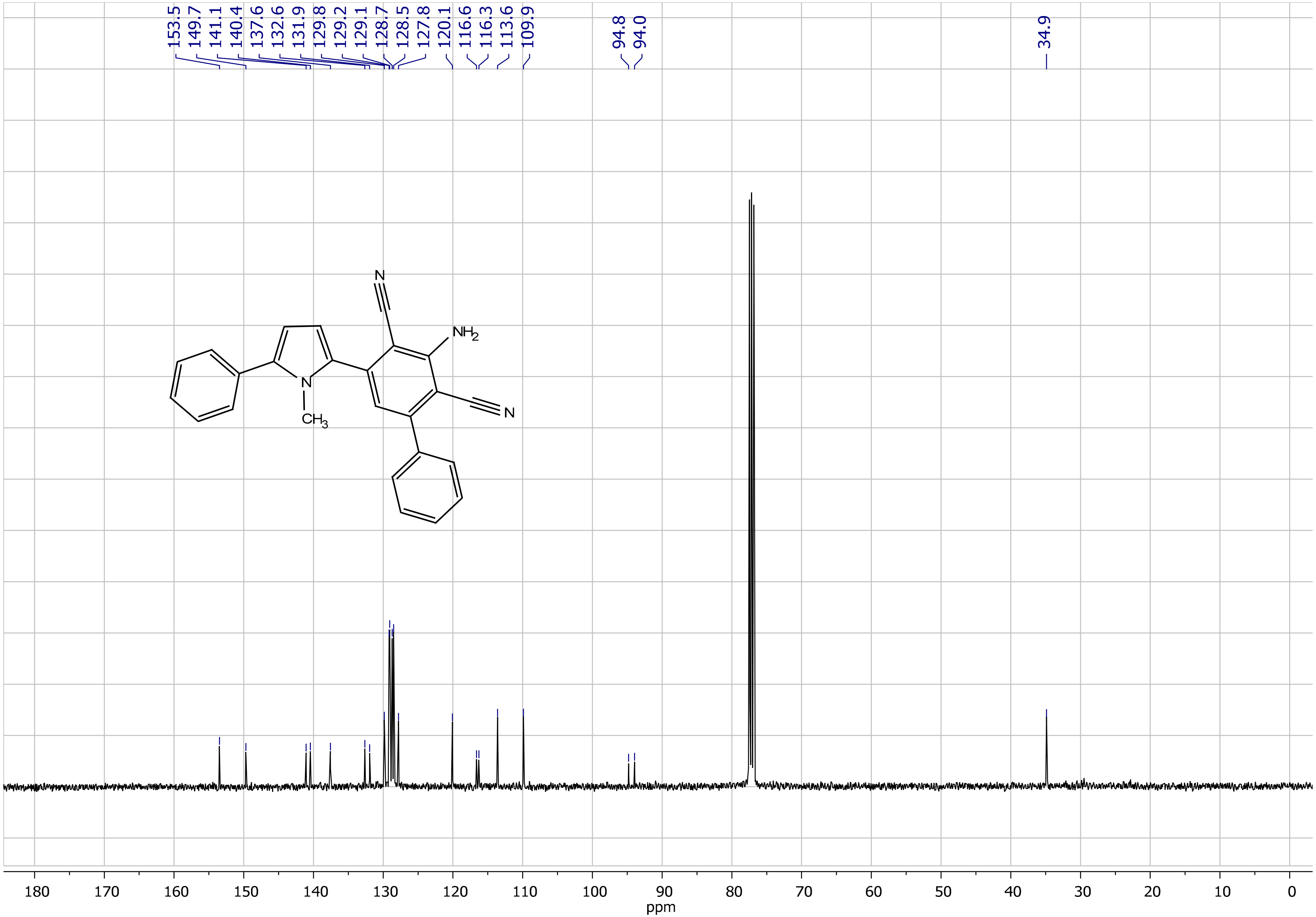

^{1}H NMR spectrum of 3-amino-5-(1-benzyl-5-phenyl-1*H*-pyrrol-2-yl)-[1,1'-biphenyl]-2,4-dicarbonitrile (**3k**) in $CDCl_3$.

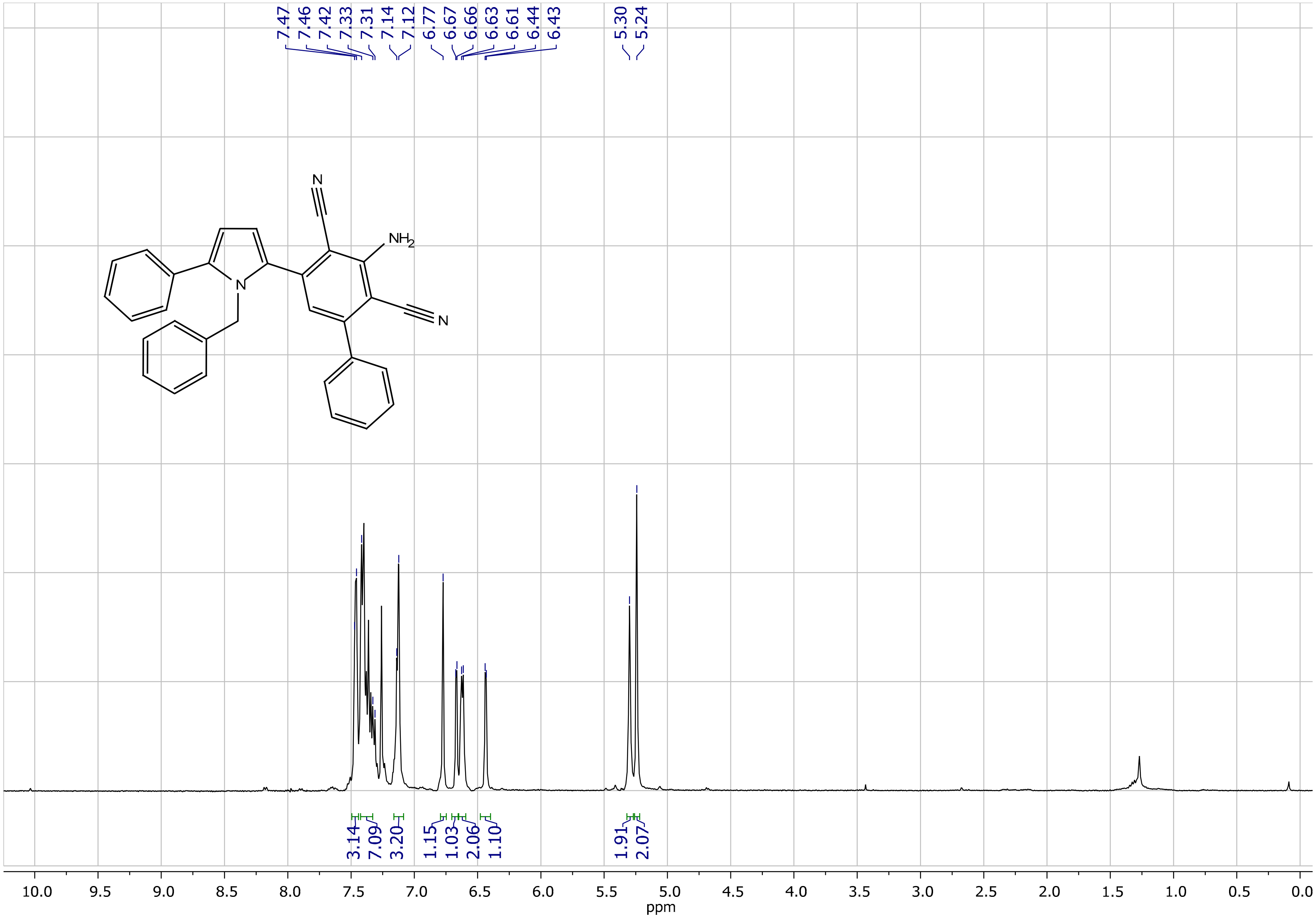

^{13}C NMR spectrum of 3-amino-5-(1-benzyl-5-phenyl-1*H*-pyrrol-2-yl)-[1,1'-biphenyl]-2,4-dicarbonitrile (**3k**) in $CDCl_3$.

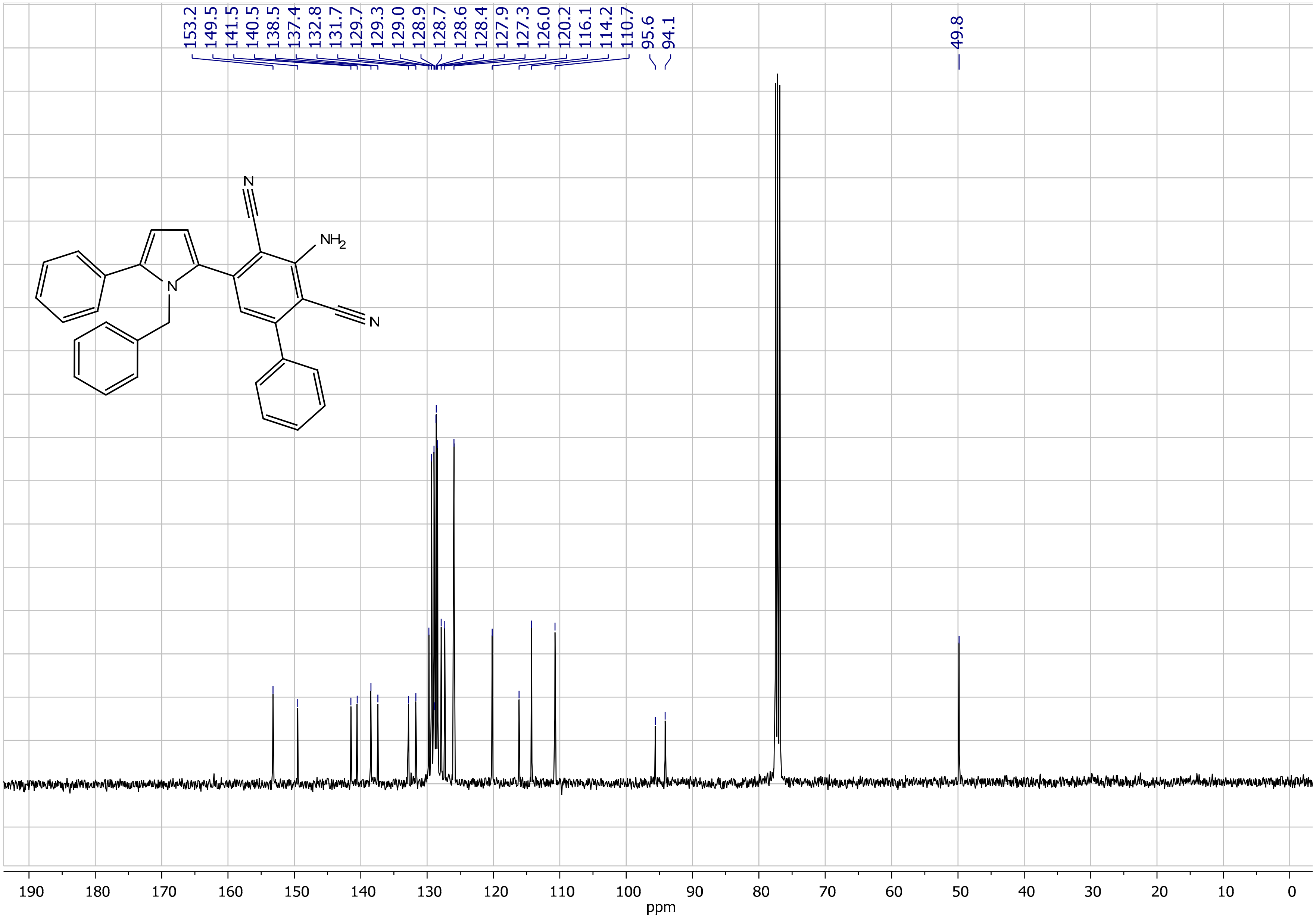

^{1}H NMR spectrum of 2-amino-4-(1-benzyl-5-phenyl-1*H*-pyrrol-2-yl)-6-(furan-2-yl)isophthalonitrile (**3l**) in $CDCl_3$.

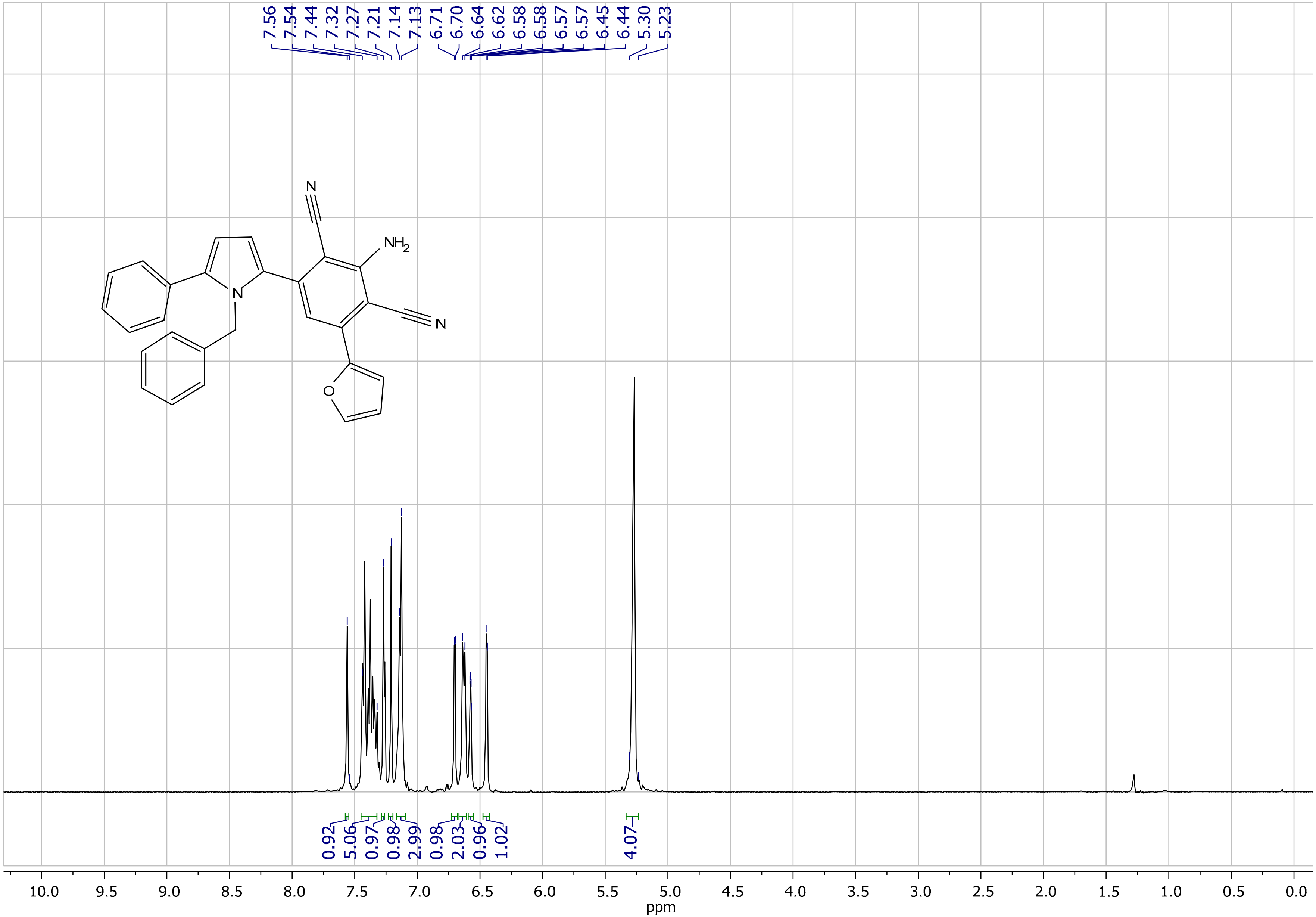

^{13}C NMR spectrum of 2-amino-4-(1-benzyl-5-phenyl-1*H*-pyrrol-2-yl)-6-(furan-2-yl)isophthalonitrile (**3l**) in $CDCl_3$.

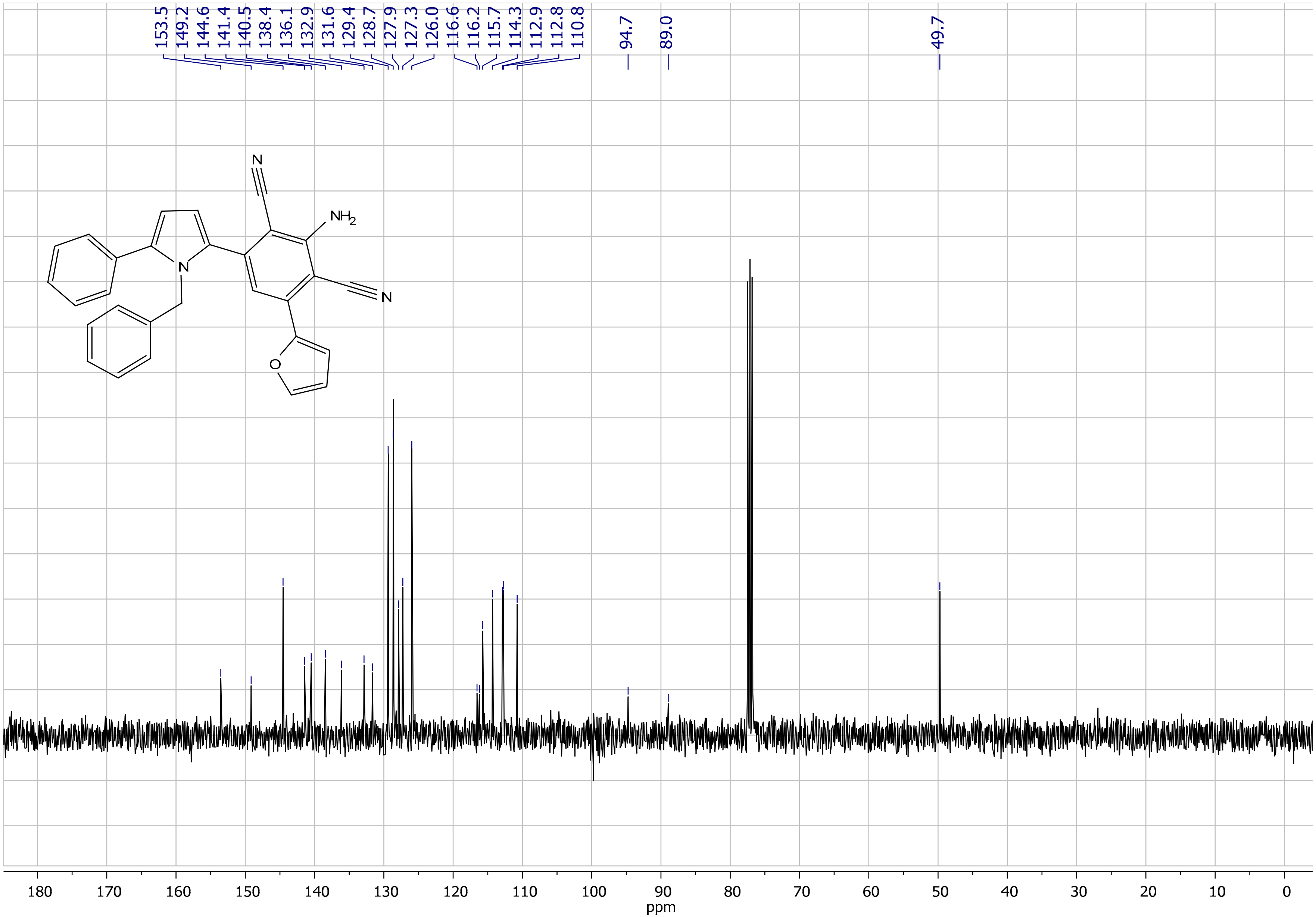

^{1}H NMR spectrum of 2-amino-4-(1-benzyl-5-phenyl-1*H*-pyrrol-2-yl)-6-(thiophen-2-yl)isophthalonitrile (**3m**) in $CDCl_3$.

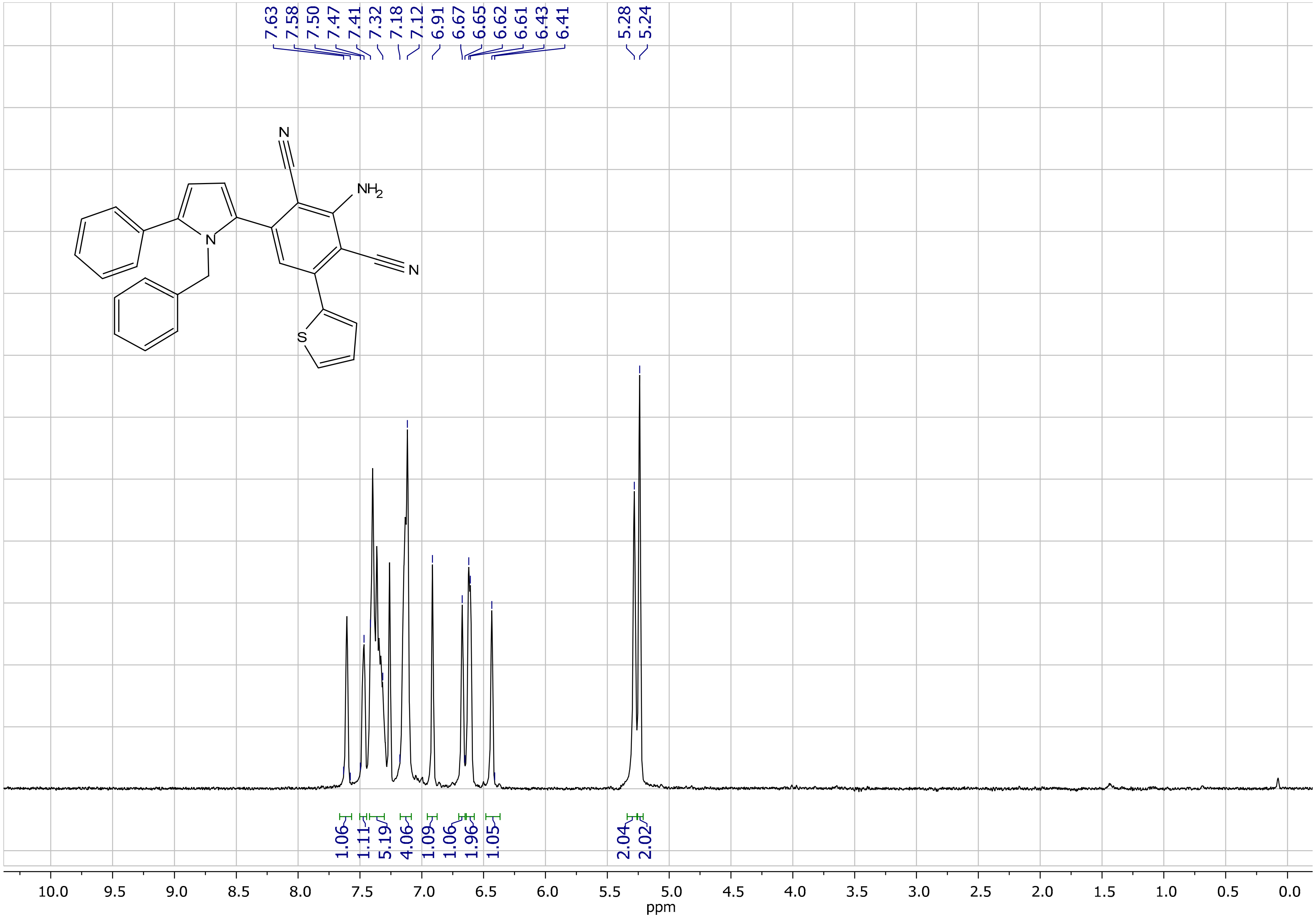

^{13}C NMR spectrum of 2-amino-4-(1-benzyl-5-phenyl-1*H*-pyrrol-2-yl)-6-(thiophen-2-yl)isophthalonitrile (**3m**) in $CDCl_3$.

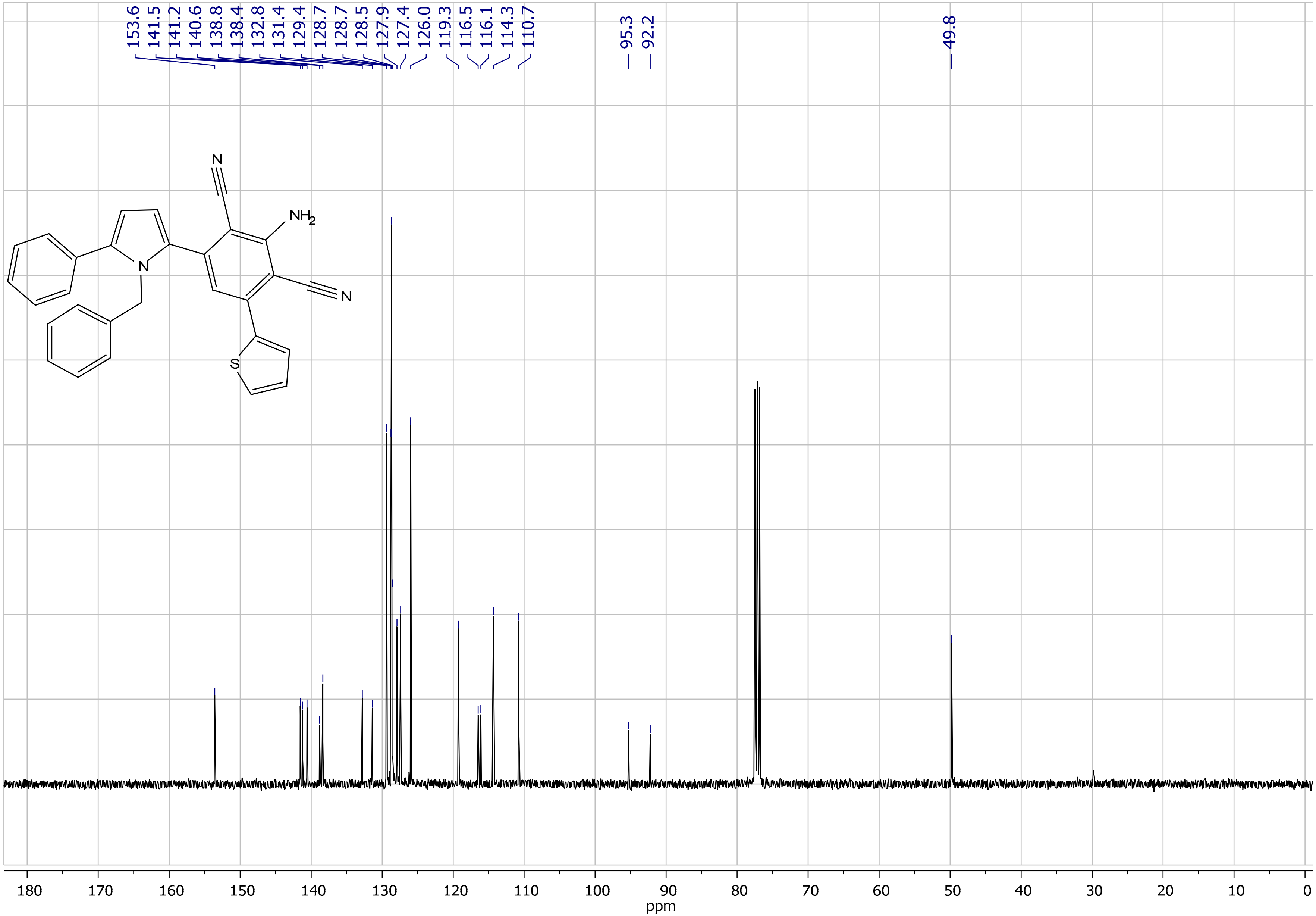

^{1}H NMR spectrum of (*Z*)-2-(3-hydroxy-3-phenyl-1-(1*H*-pyrrol-2-yl)allylidene)malononitrile (**4a**) in DMSO-d$_6$.

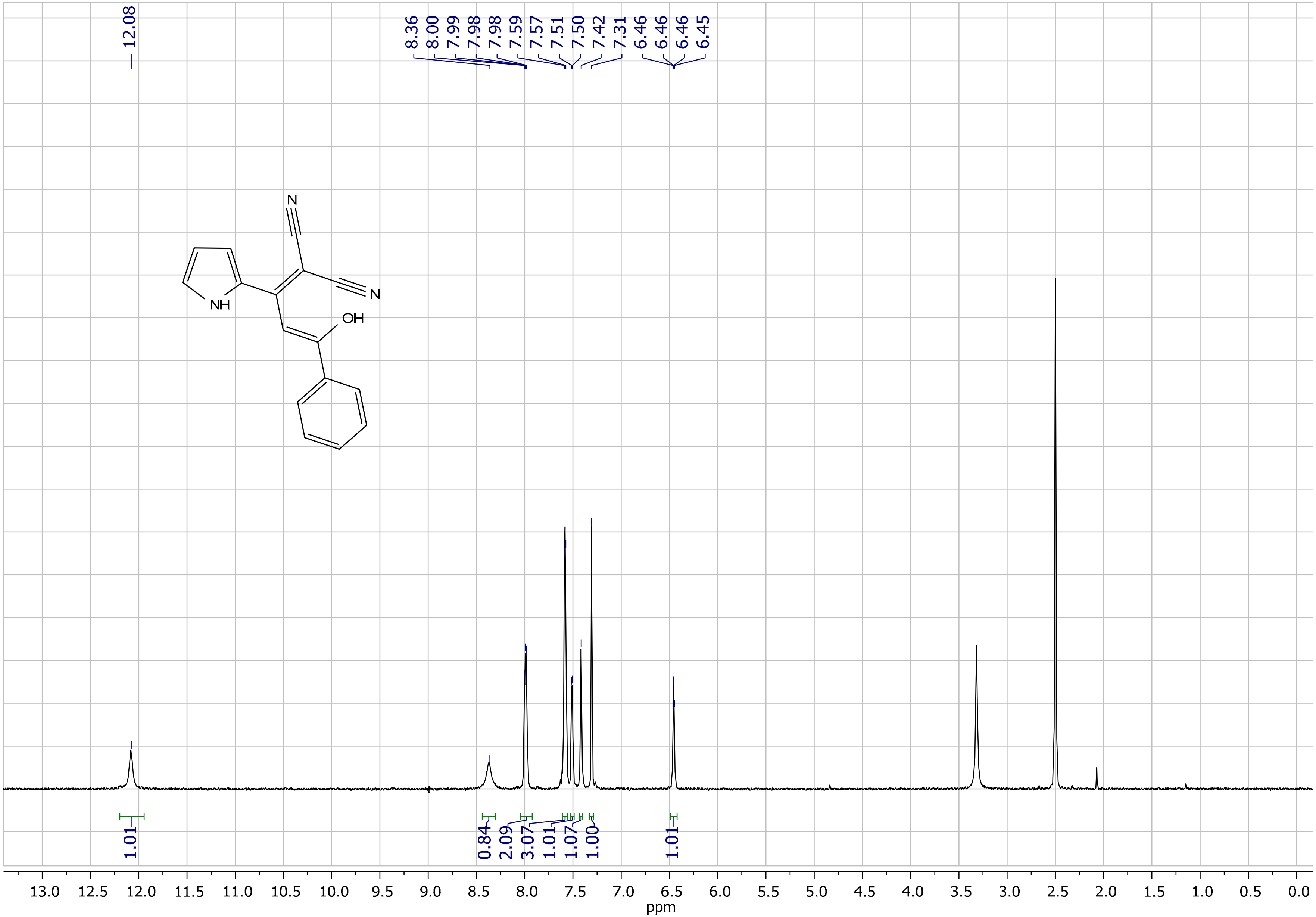

^{13}C NMR spectrum of (*Z*)-2-(3-hydroxy-3-phenyl-1-(1*H*-pyrrol-2-yl)allylidene)malononitrile (**4a**) in DMSO-d$_6$.

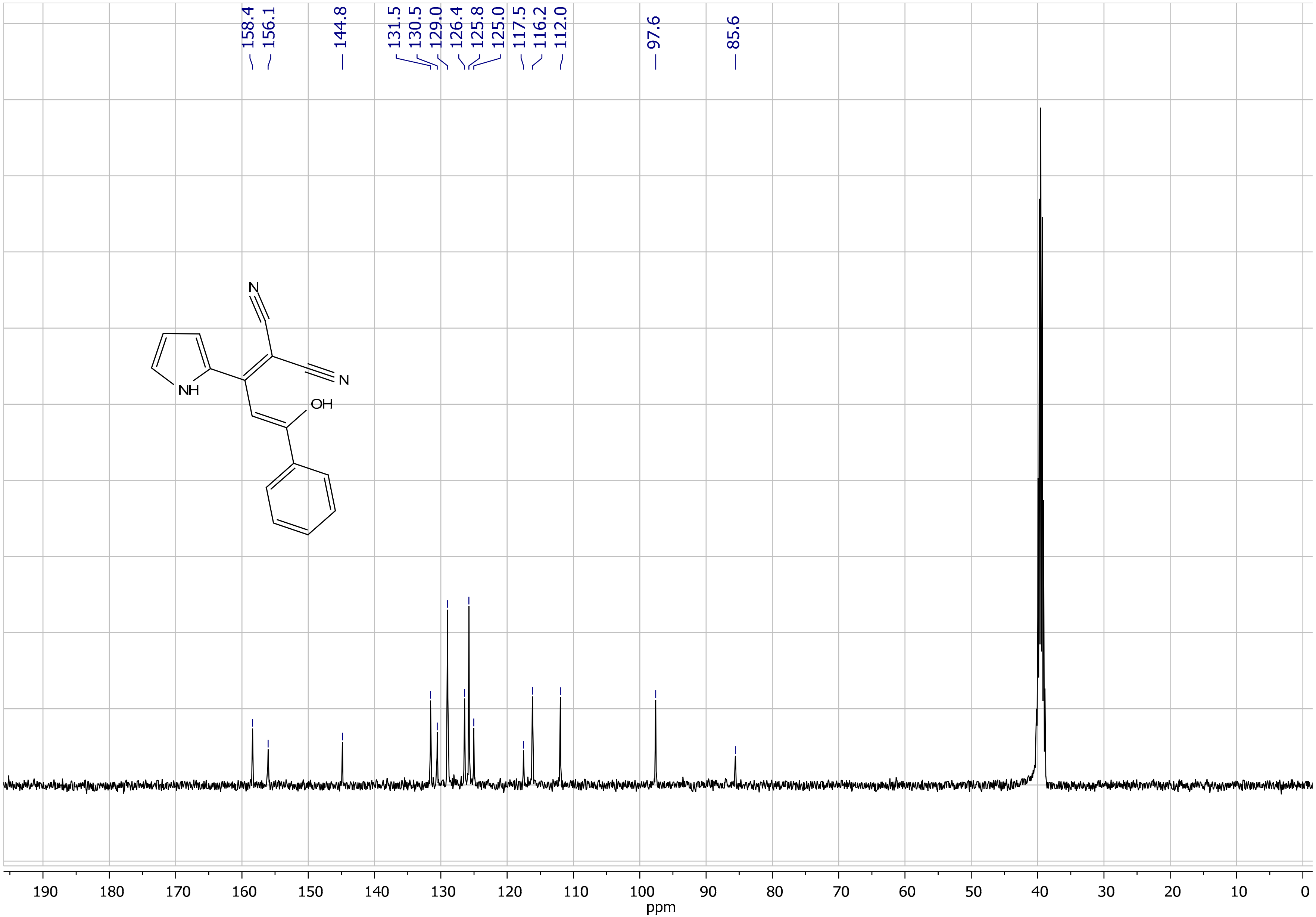

^{1}H NMR spectrum of (*Z*)-2-(3-hydroxy-3-(2-furyl)-1-(1*H*-pyrrol-2-yl)allylidene)malononitrile (**4b**) in DMSO-d$_6$.

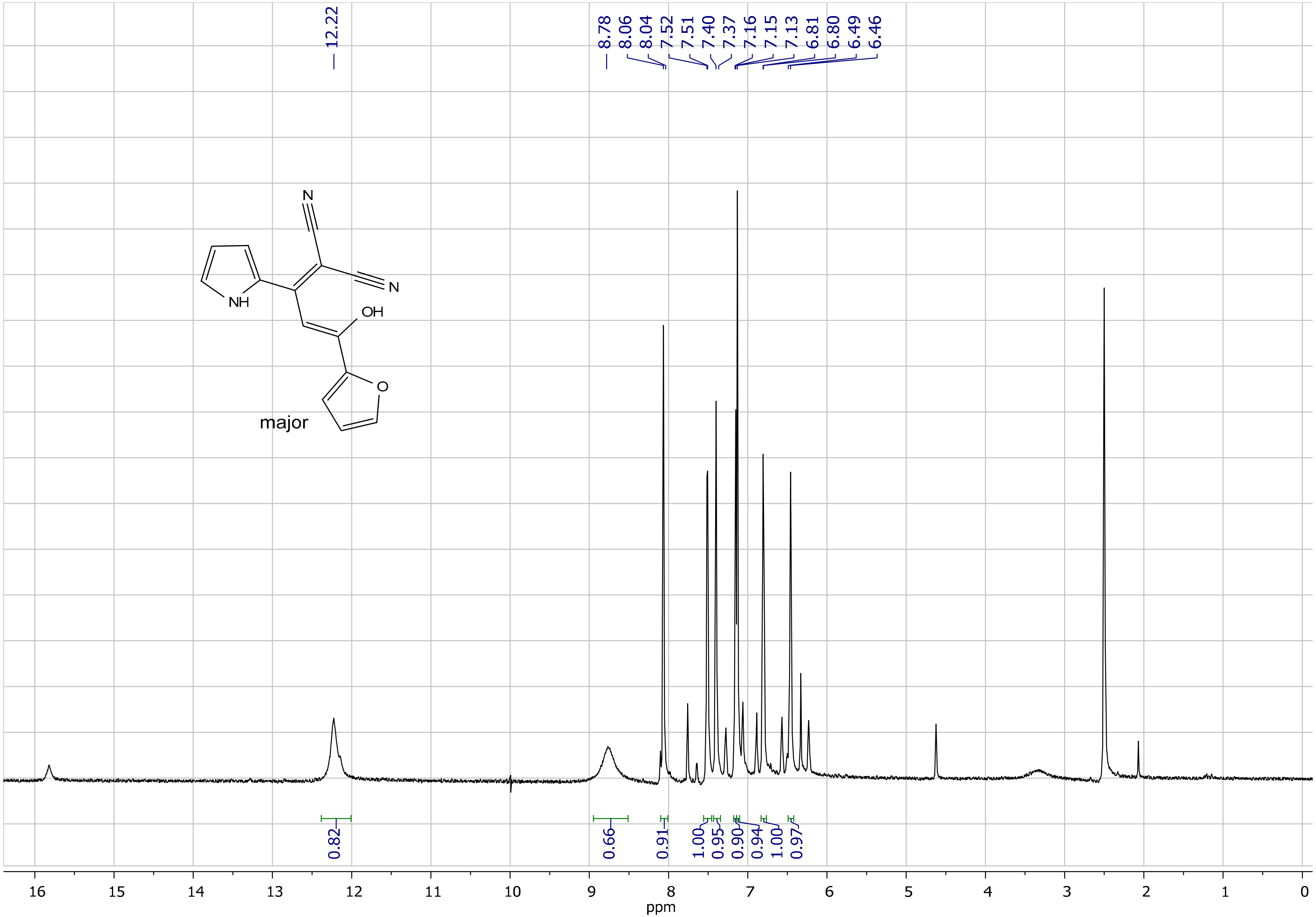

^{1}H NMR spectrum of impurity in **4b**, the keto form of **4b** in DMSO-d_6.

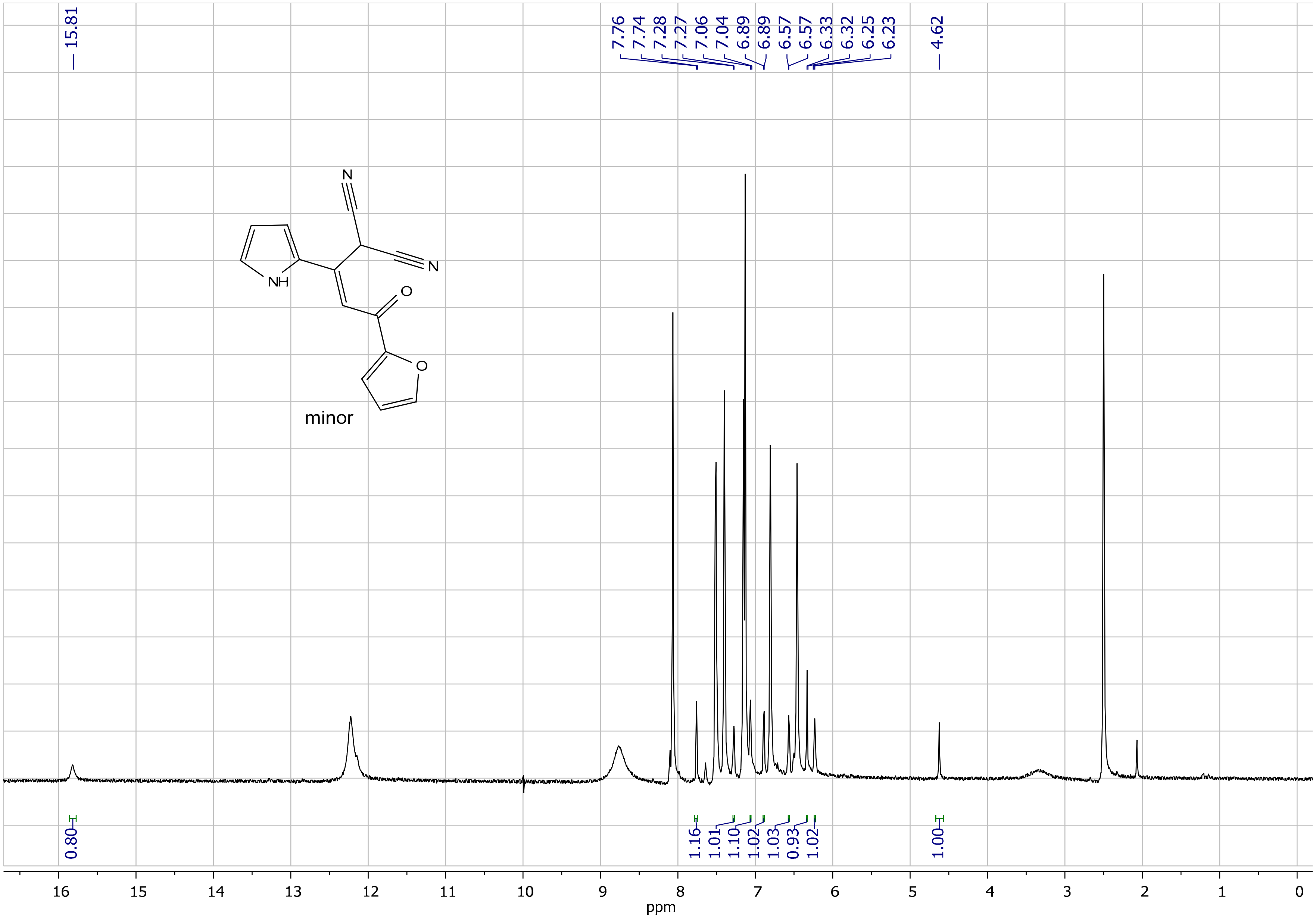

^{1}H NMR spectrum of ((*Z*)-2-(3-Hydroxy-1-(1*H*-pyrrol-2-yl)-3-(thiophen-2-yl)allylidene)malononitrile (**4c**) in DMSO-d_6.

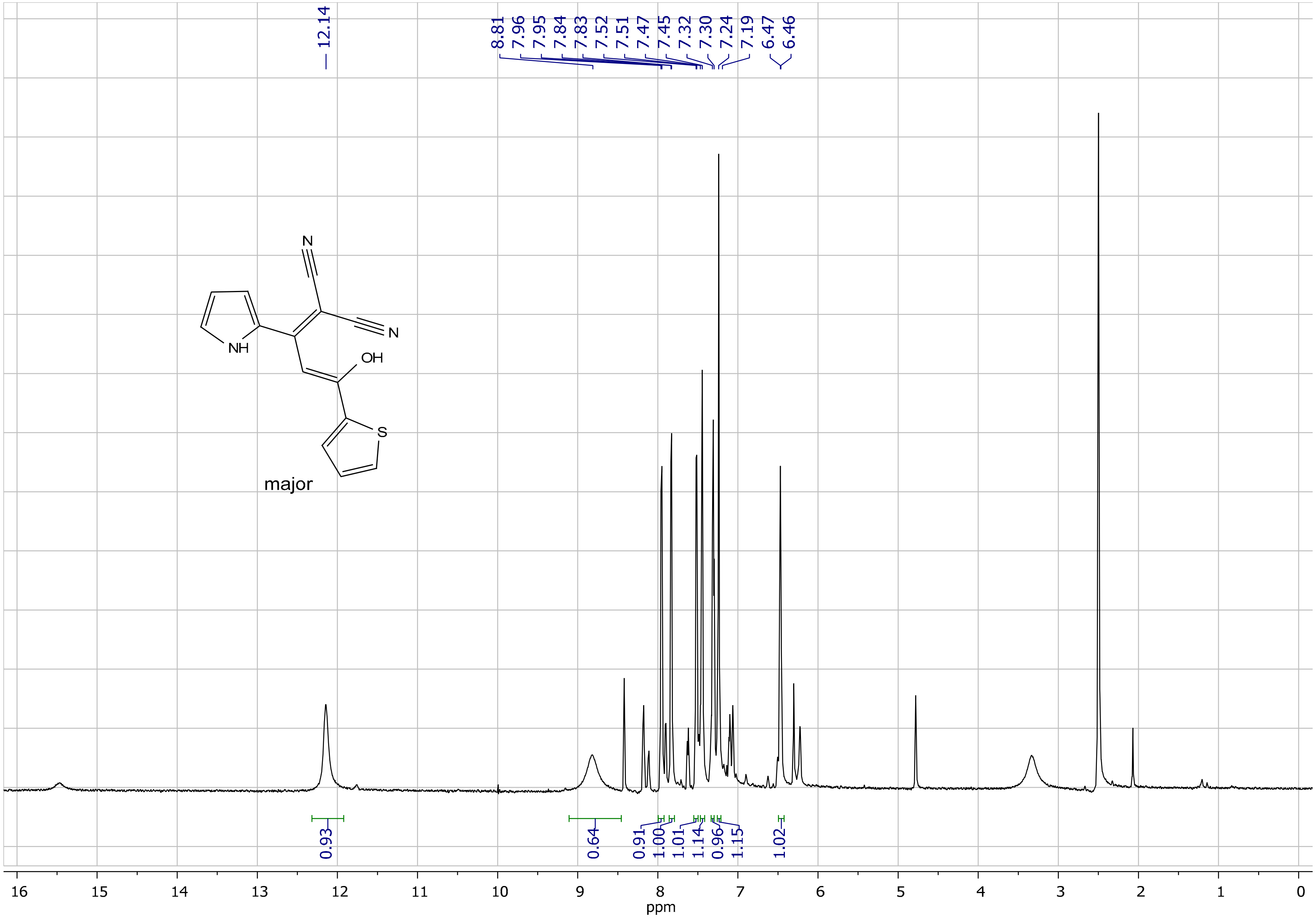

^{13}C NMR spectrum of ((*Z*)-2-(3-Hydroxy-1-(1*H*-pyrrol-2-yl)-3-(thiophen-2-yl)allylidene)malononitrile (**4c**) in DMSO-d$_6$.

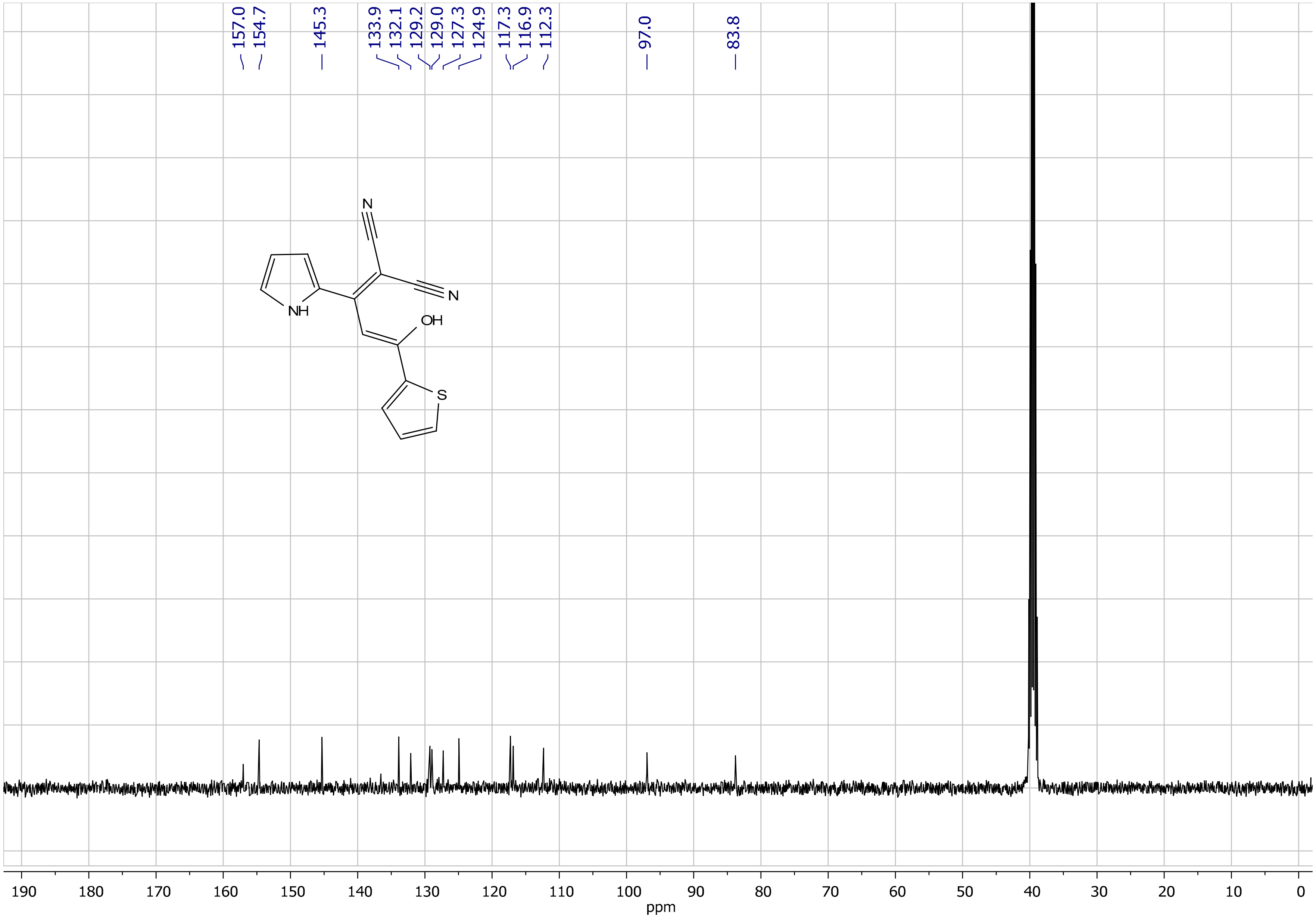

^{1}H NMR spectrum of impurity in **4c**, the keto form of **4c** in DMSO-d_6.

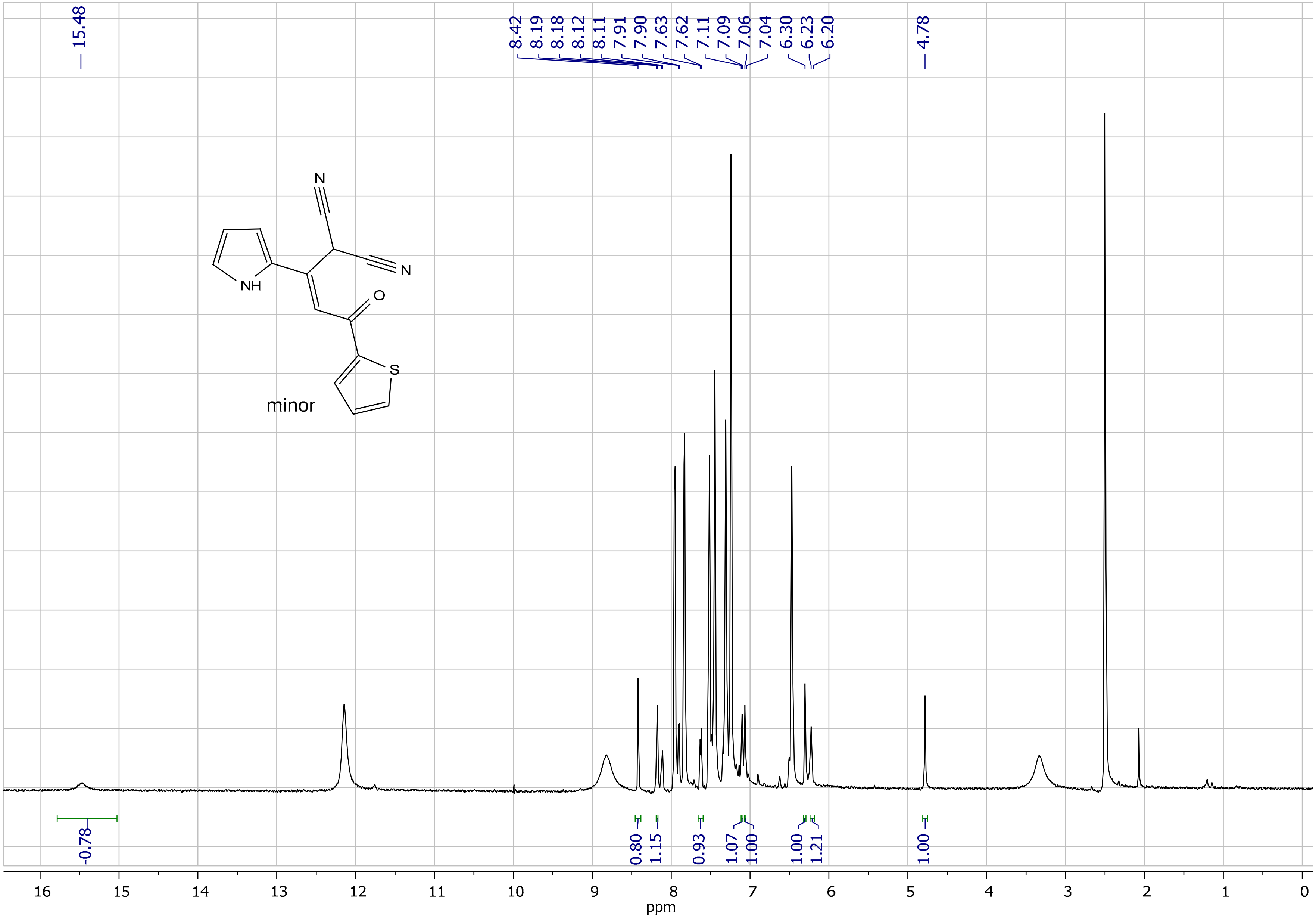

^{1}H NMR spectrum of (*Z*)-3-amino-1-(2-oxo-2-phenylethylidene)-1*H*-pyrrolizine-2-carbonitrile (**6a**) in DMSO-d$_6$.

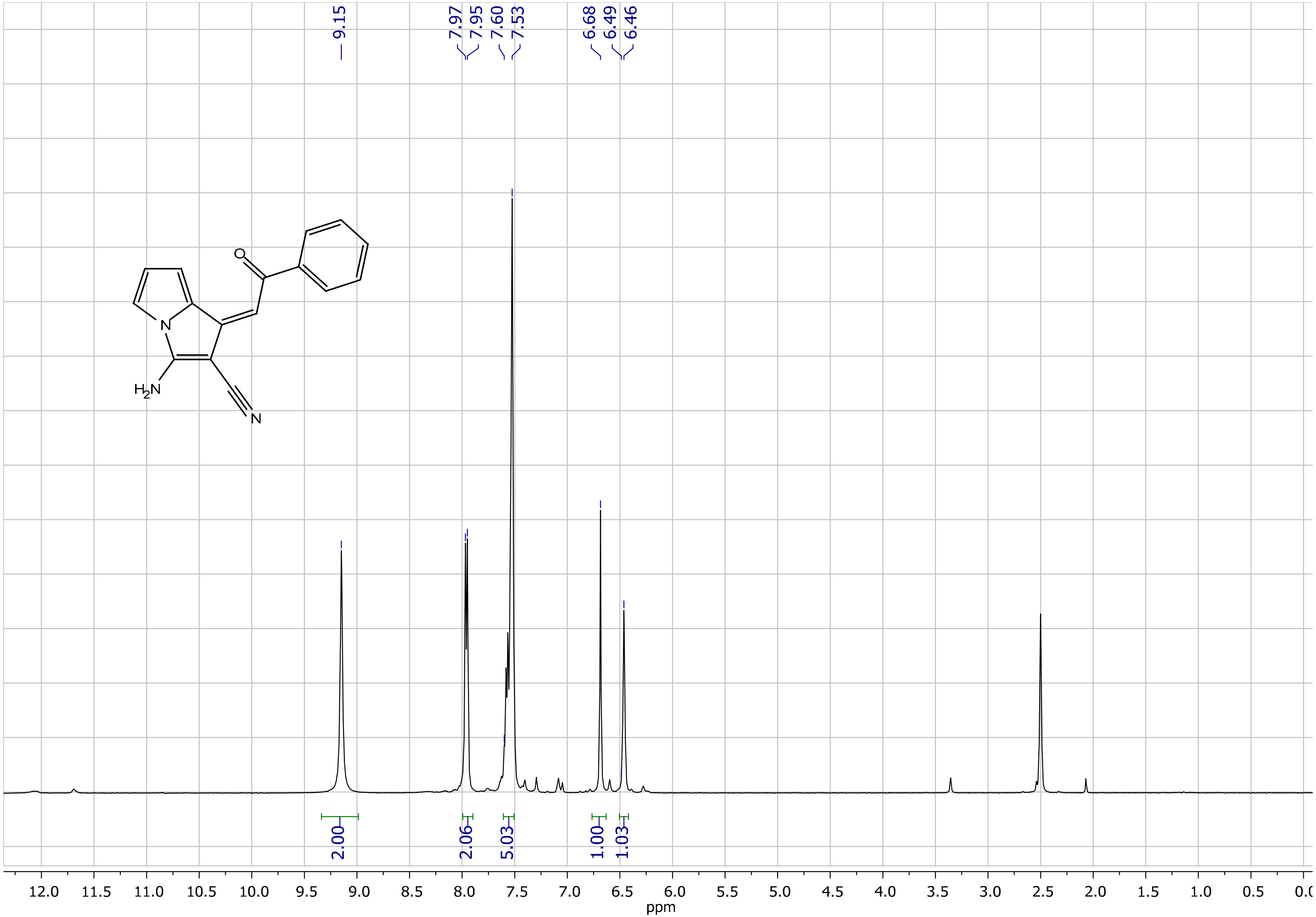

^{13}C NMR spectrum of (*Z*)-3-amino-1-(2-oxo-2-phenylethylidene)-1*H*-pyrrolizine-2-carbonitrile (**6a**) in DMSO-d$_6$.

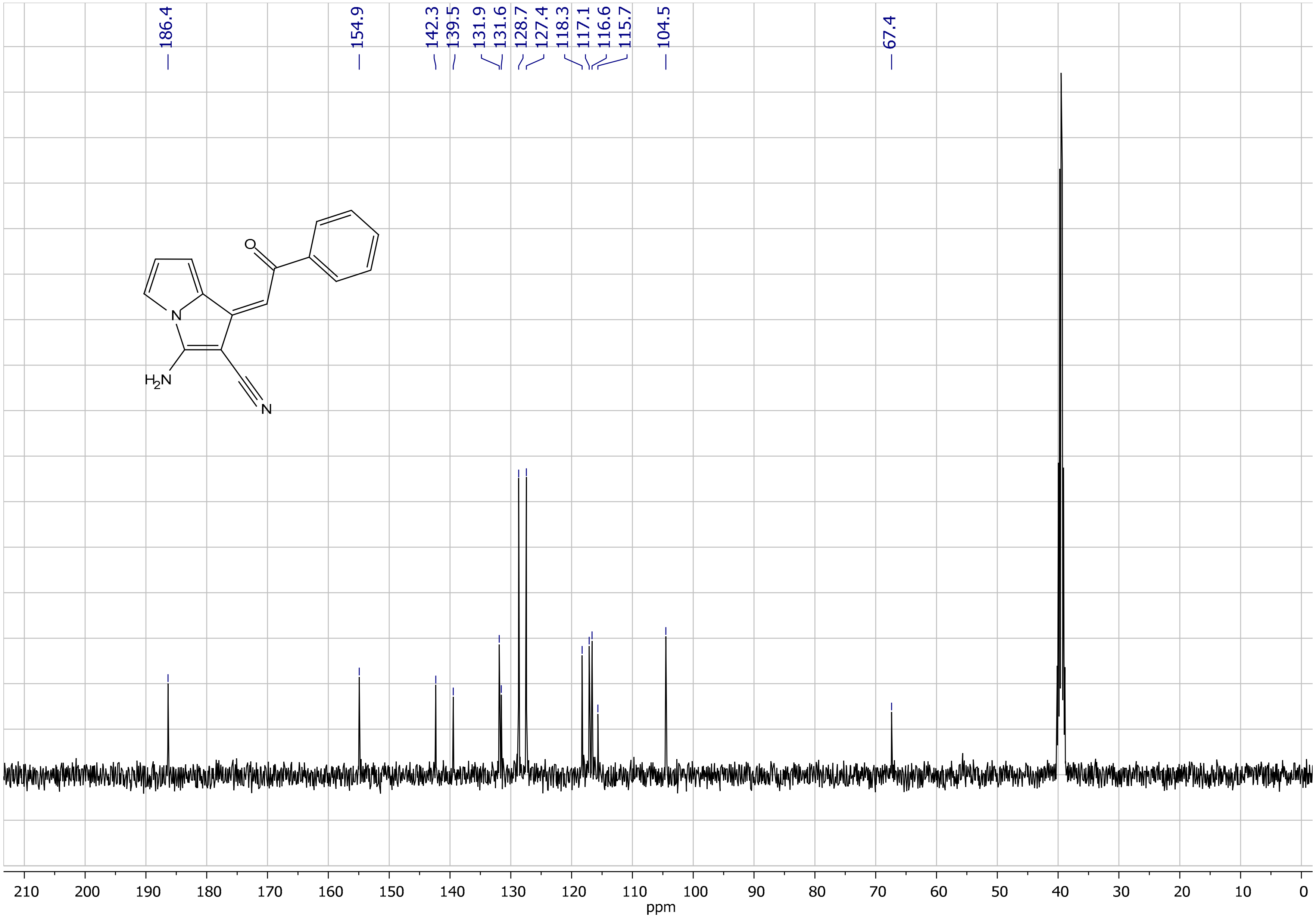

^{1}H NMR spectrum of (*Z*)-3-amino-1-(2-(furan-2-yl)-2-oxoethylidene)-1*H*-pyrrolizine-2-carbonitrile (**6b**) in DMSO-d$_6$.

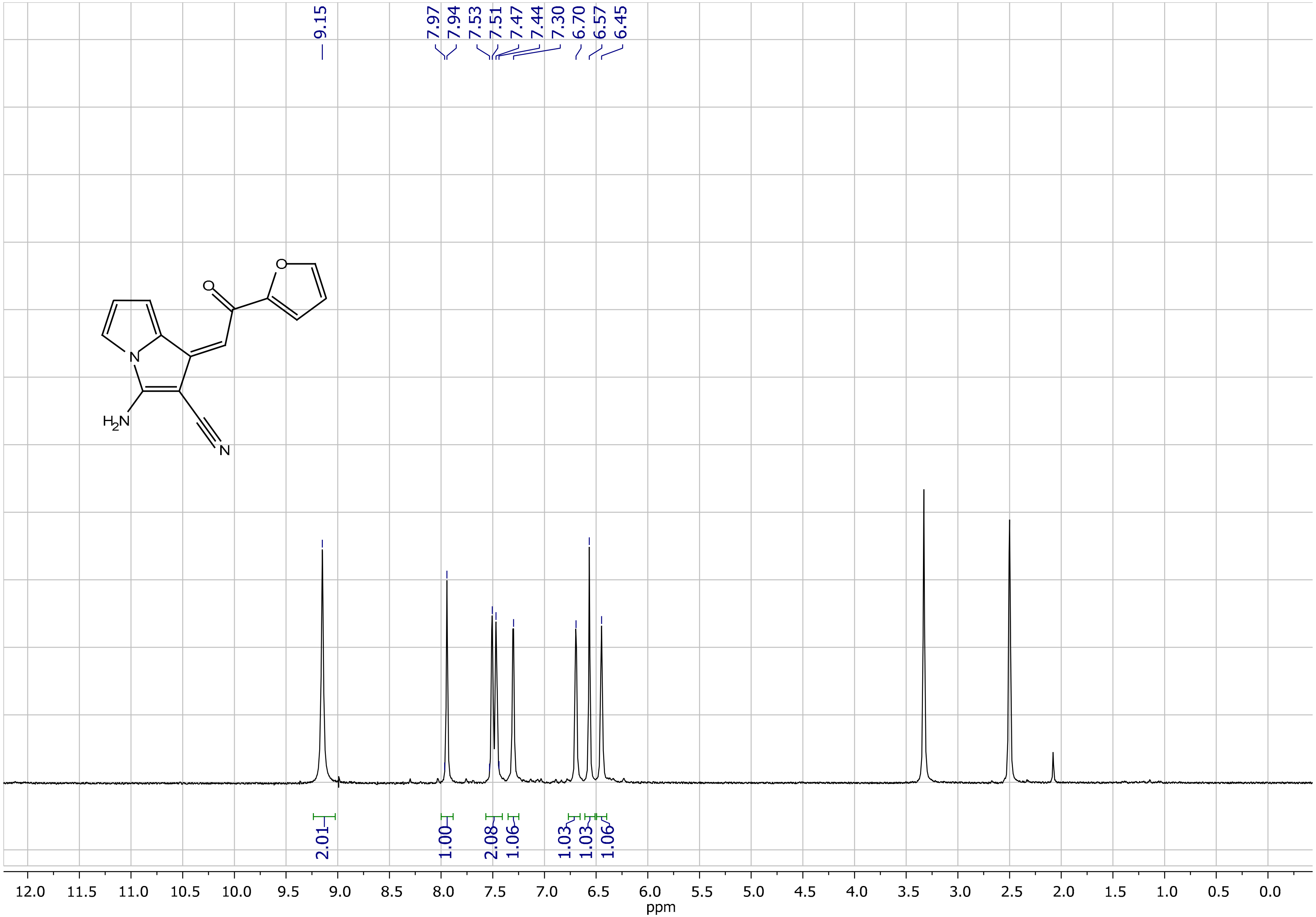

^{13}C NMR spectrum of (*Z*)-3-amino-1-(2-(furan-2-yl)-2-oxoethylidene)-1*H*-pyrrolizine-2-carbonitrile (**6b**) in DMSO-d$_6$.

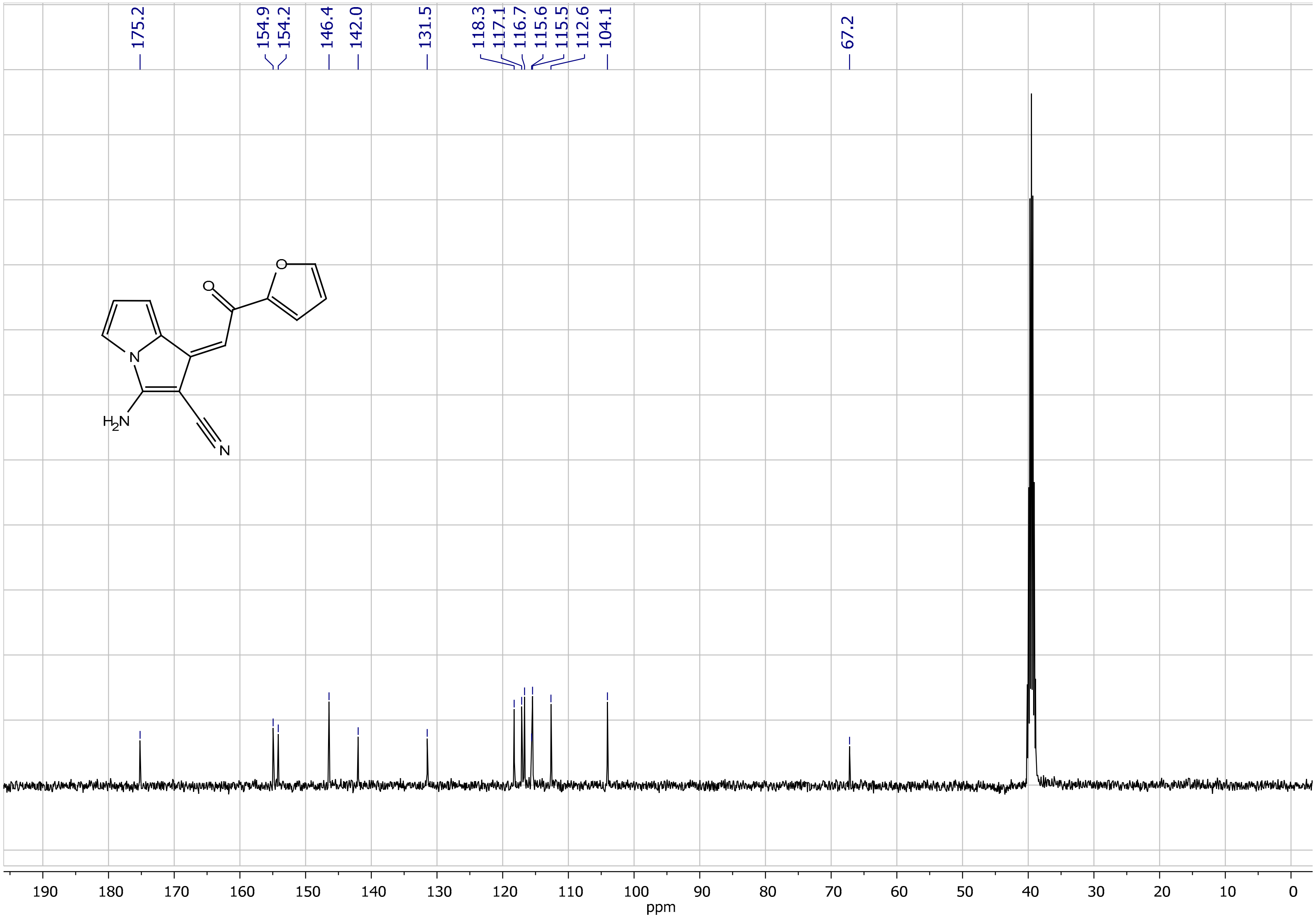

^{1}H NMR spectrum of (*Z*)-3-amino-1-(2-oxo-2-(thiophen-2-yl)ethylidene)-1*H*-pyrrolizine-2-carbonitrile (**6c**) in DMSO-d_6.

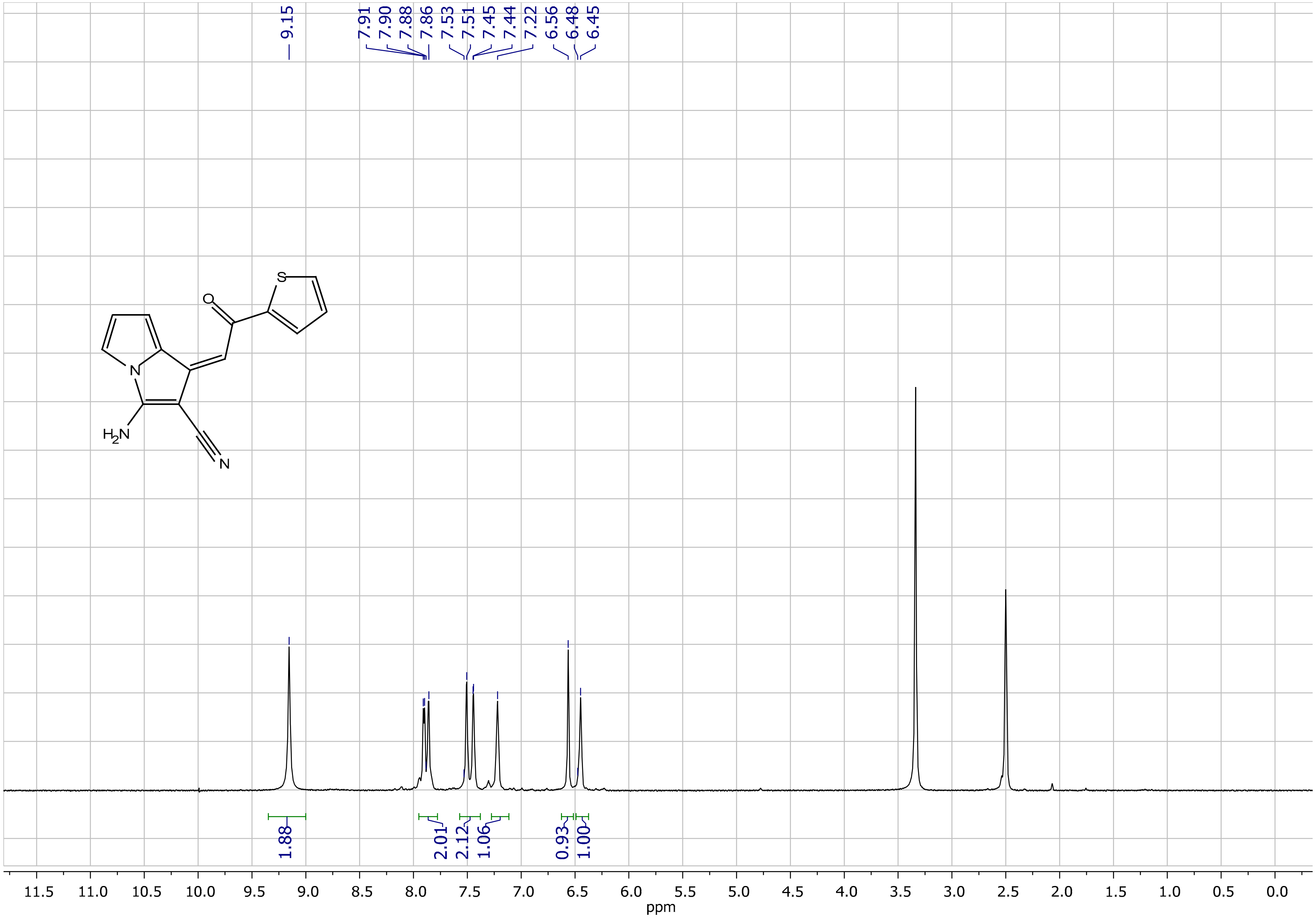

^{13}C NMR spectrum of (*Z*)-3-amino-1-(2-oxo-2-(thiophen-2-yl)ethylidene)-1*H*-pyrrolizine-2-carbonitrile (**6c**) in DMSO-d$_6$.

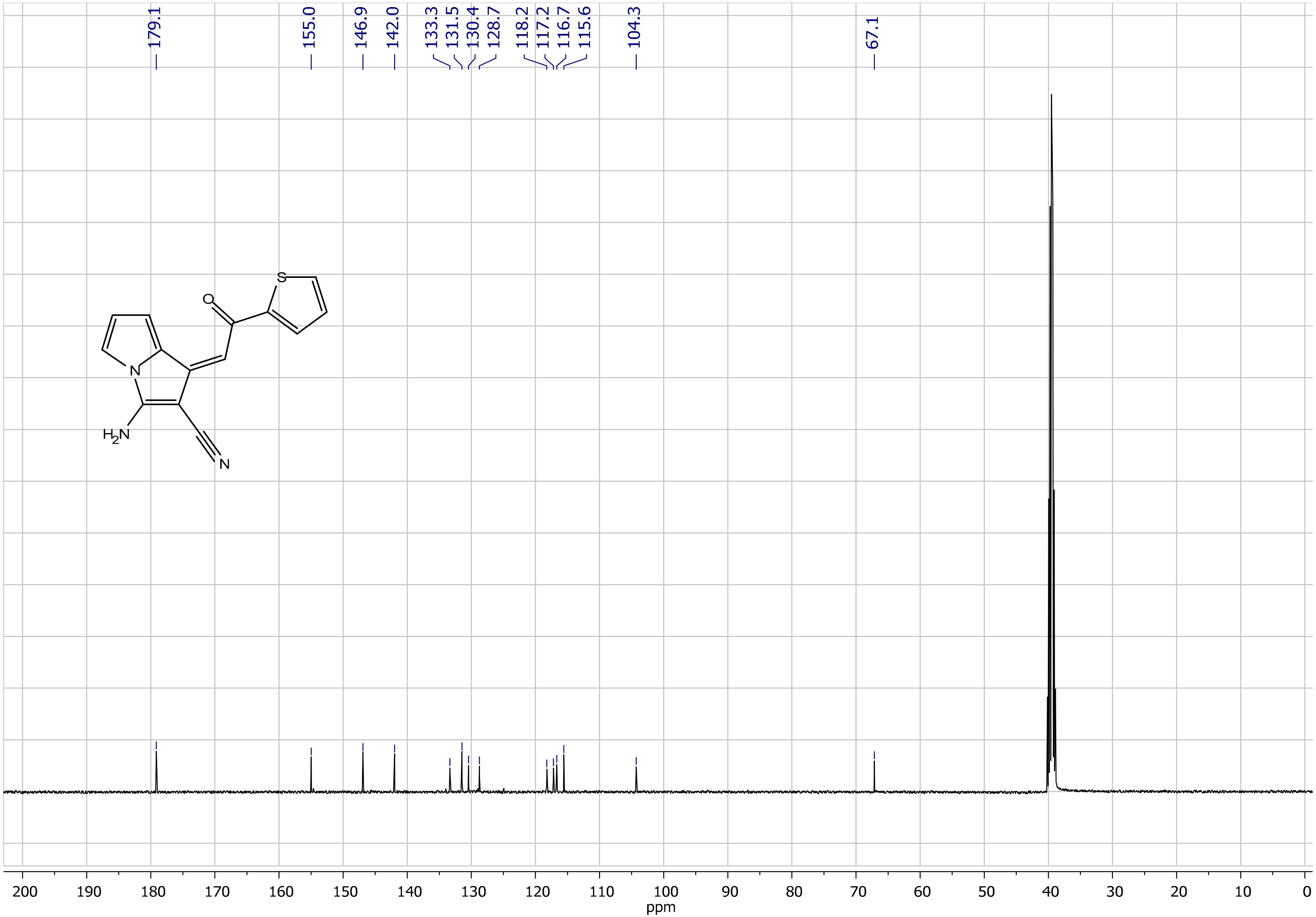

The 2D NMR spectra of aminopyrrolizine 6c:

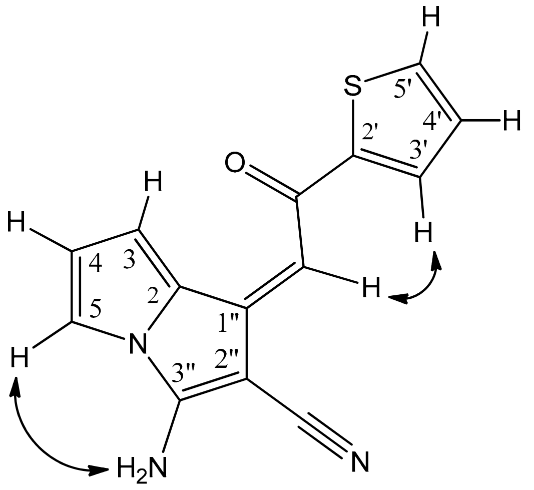

Z - isomer

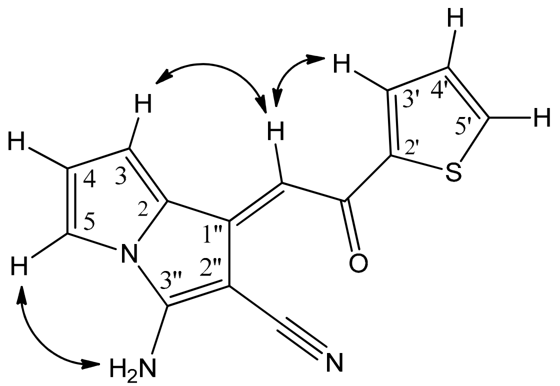

E - isomer

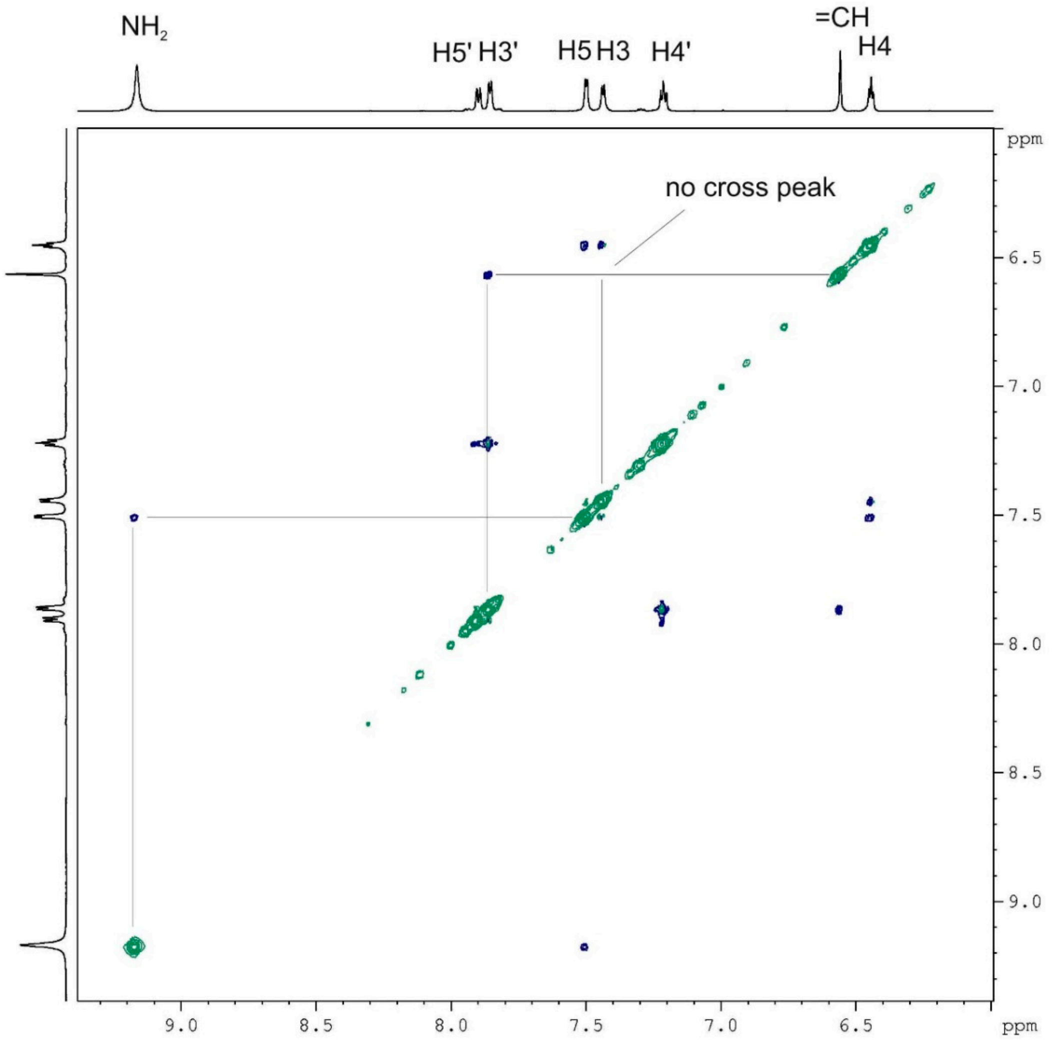

2D NOESY NMR spectrum of aminopyrrolizine **6c** (DMSO-d_6). The position, where should be a cross-peak between the signals =CH and H3 proton in the case of the *E*-isomer, is shown.

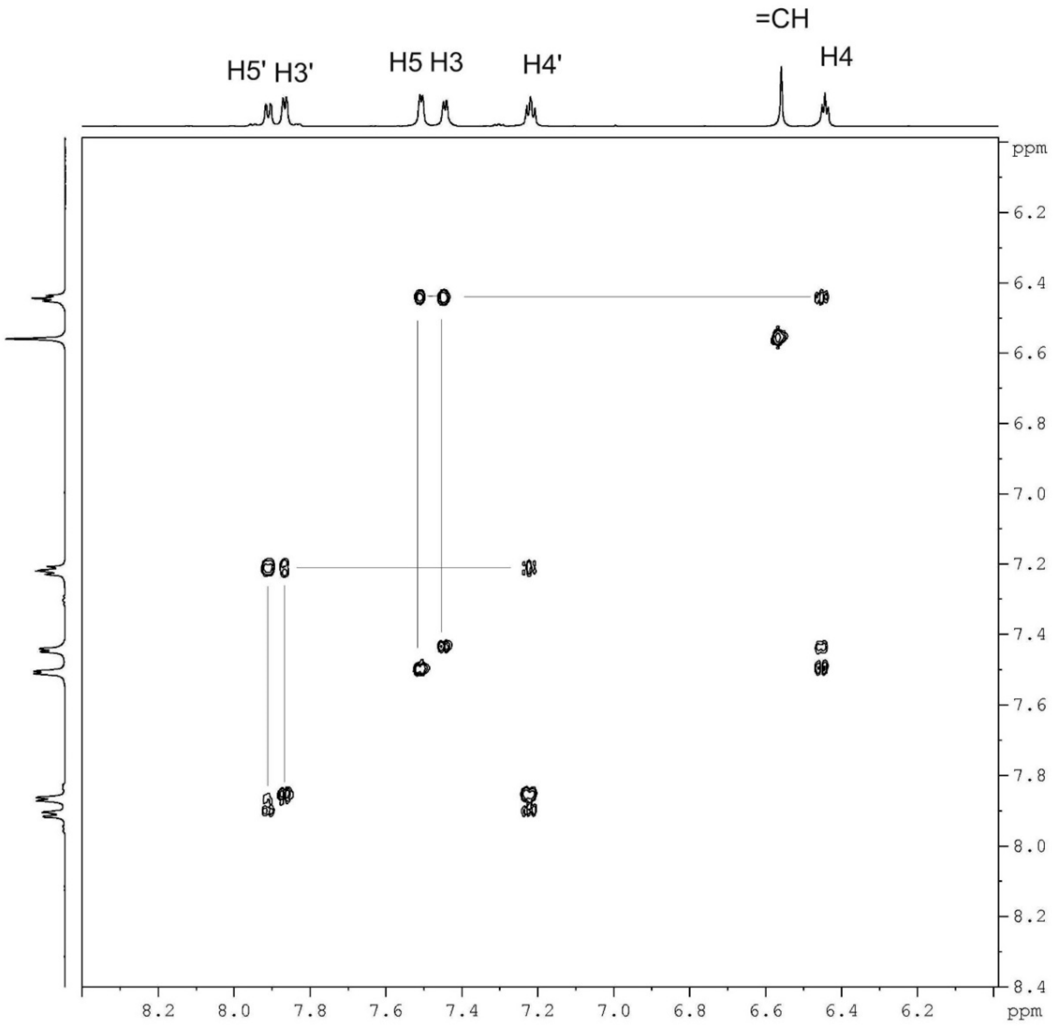

2D COSY NMR spectrum of **6c** (DMSO-d_6). The assignment of the spin systems of thiophene and pyrrole is shown.

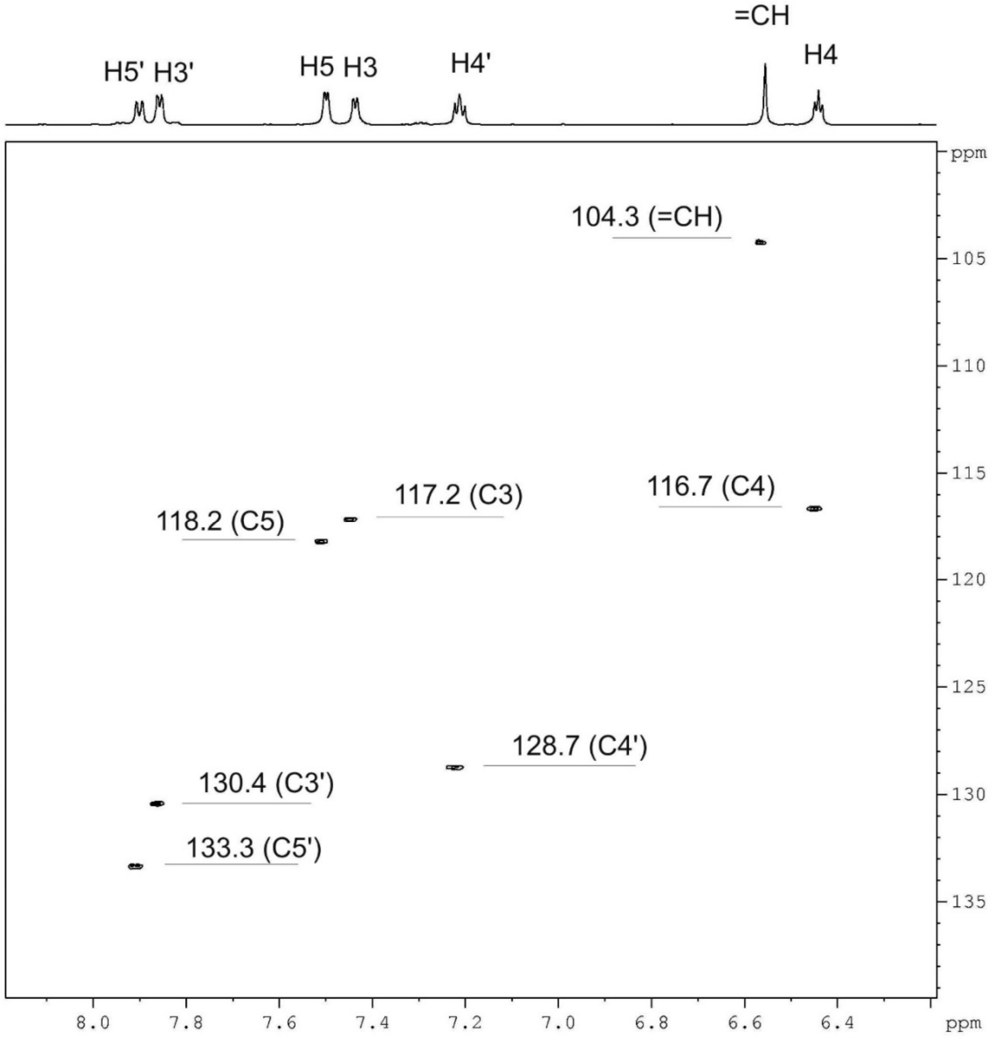

2D ^{1}H-^{13}C HSQC NMR spectrum of **6c** (DMSO-d_6). The assignment of carbon signals directly associated with protons in the spectrum ^{13}C NMR is shown.

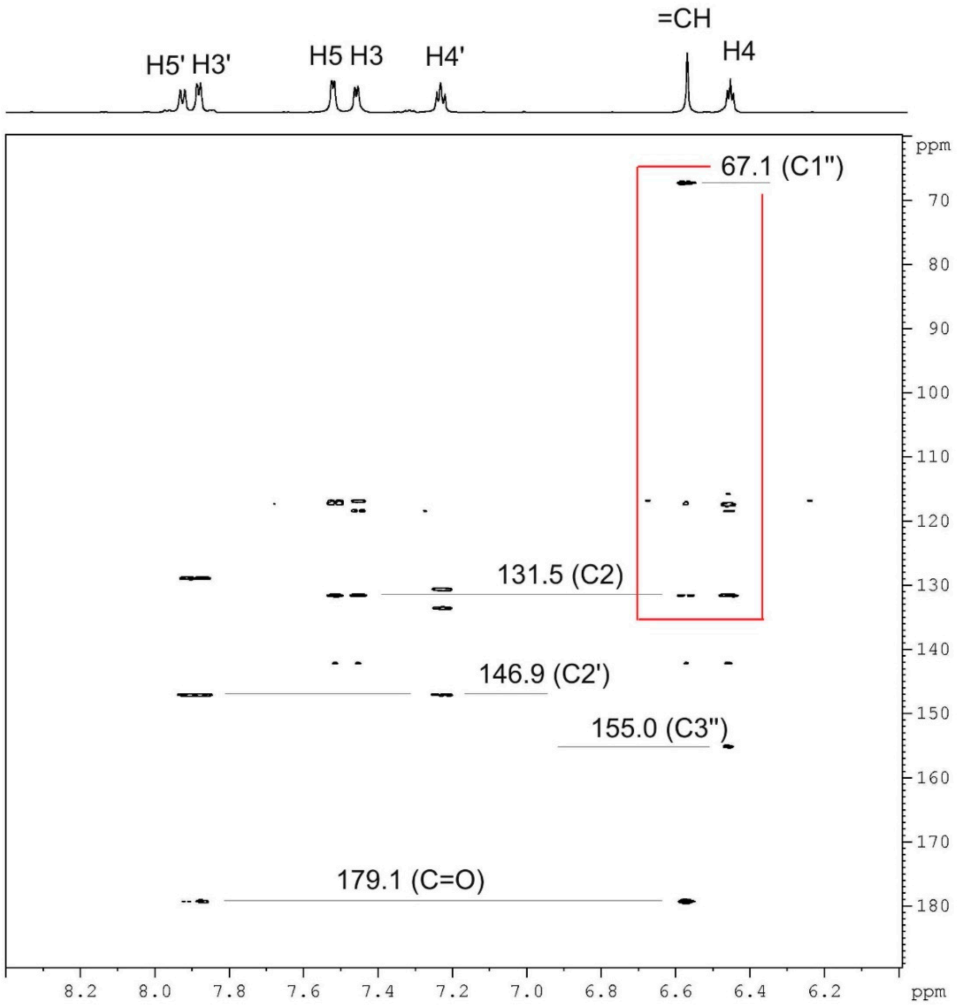

2D ^{1}H-^{13}C HMBC NMR spectrum of **6c** (DMSO-d_6). The assignment of quaternary carbon atoms in the ^{13}C NMR spectrum is shown. The region of the spectrum (next figure) is selected, according to which the values of the vicinal constants of the spin-spin interaction ^{3}J(C,H) were measured.

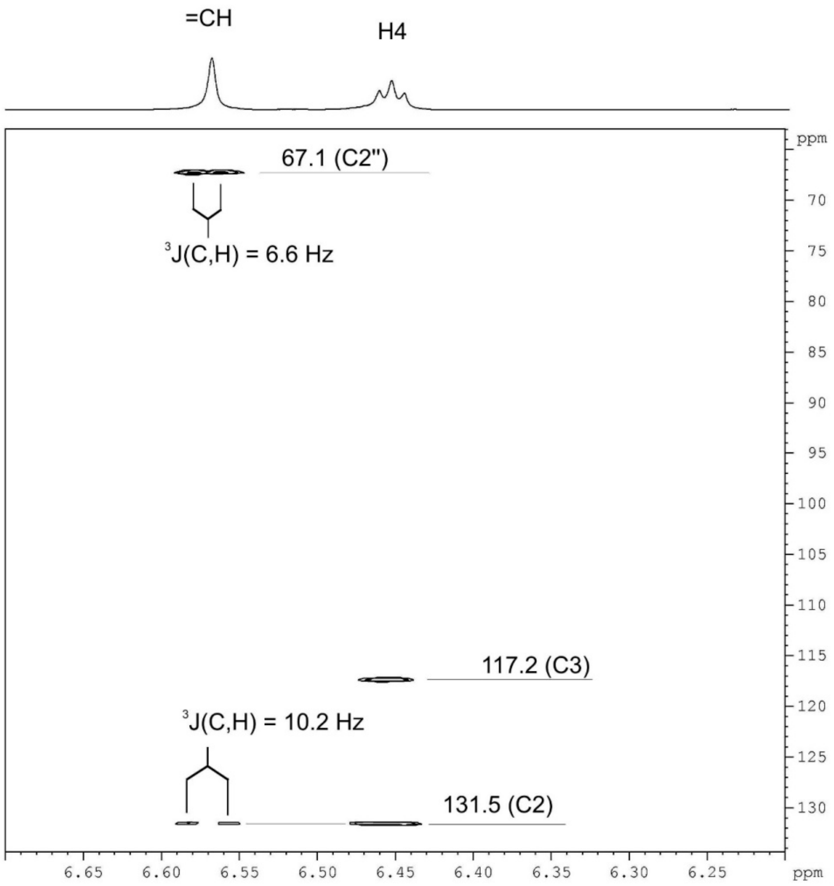

Fragment of 2D ^{1}H-^{13}C HMBC NMR spectrum of **6c** (DMSO-d_6). The values of the constants and cross-peaks from the proton signal =CH are shown, along the cross-sections of which the values of the vicinal constants of the spin-spin interaction were measured ^{3}J(C,H). Value ^{3}J(C2,H) > ^{3}J(C2'',H) corresponding to the stereospecific dependence ^{3}J(*trans*) > ^{3}J(*cis*) confirms the Z-configuration of the connection 6c.

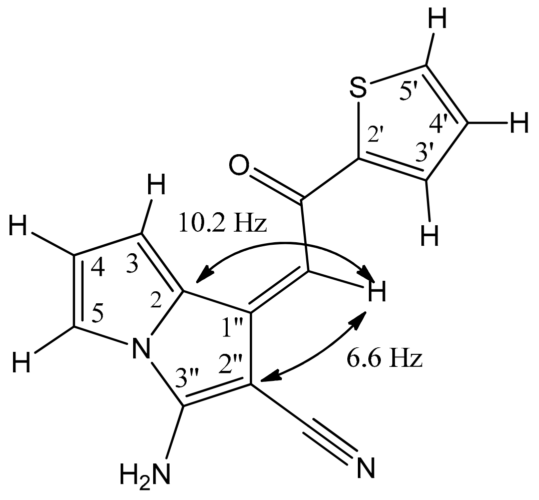

Z - isomer
